# Using ecological niche modelling to prioritise areas for conservation of the critically endangered Buffy‐Headed marmoset (*Callithrix flaviceps*)

**DOI:** 10.1002/ece3.11203

**Published:** 2024-04-05

**Authors:** Léa Bataillard, Ane Eriksen, Fabiano R. de Melo, Adriana Pereira Milagres, Olivier Devineau, Orlando Vítor Vital

**Affiliations:** ^1^ Department of Forestry and Wildlife Management Inland Norway University of Applied Sciences Koppang Norway; ^2^ Department of Forestry Engineering Federal University of Viçosa Viçosa Minas Gerais Brazil

**Keywords:** Atlantic forest, *Callithrix flaviceps*, climate change, critically endangered primates, habitat suitability, niche modelling

## Abstract

Endemic to the Atlantic Forest in Southeastern Brazil, the critically endangered Buffy‐Headed marmoset (*Callithrix flaviceps*) is lacking the required attention for effective conservation. We modelled its ecological niche with the main objectives of (1) defining suitable habitat and (2) prioritising areas for conservation and/or restoration. The current geographical range of *Callithrix flaviceps* in the Atlantic Forest of Southeast Brazil. We used Ensemble Species Distribution Modelling to define current habitat suitability considering four climate and two landscape variables. To identify areas to prioritise for conservation and/or restoration, we predicted future habitat suitability considering the intermediate (RCP4.5) and extreme (RCP8.5) climate change scenarios for the years 2050 and 2070. Among the variables included to predict current species distribution, tree canopy cover, precipitation seasonality and temperature seasonality were the most important whereas digital elevation model and precipitation during the wettest month were the least important. *Callithrix flaviceps* was most likely to occur in areas with tree canopy cover >80%, high precipitation seasonality and temperature seasonality between 21 and 23°C. From the future suitability prediction maps, the Caparaó National Park stands out as a likely key area for the preservation of the species. Furthermore, high climatic suitability but low landscape suitability suggests that habitat restoration in ‘Serra das Torres’ (South of the current distribution area) might be a useful strategy. However, creating ecological corridors on the west side of Caparaó would be necessary to improve connectivity. More surveys within and beyond the current geographical range are required to define more precisely the distribution of the species. Our results support the notion that seasonality is important for *Callithrix flaviceps* and that as a montane species, it prefers colder environments and higher altitudes. Within both climate change scenarios, Caparaó National Park was predicted to be highly suitable, with a high probability of presence.

## Graphical abstract

Prioritising areas for conservation of the least studied marmoset species (*Callithrix flaviceps*), a critically endangered primate endemic from the Atlantic Forest, southeast Brazil. Considering both climate and landscape variables, and generating maps of future projections for habitat suitability in 2050 and 2070. Photo credit: Sarisha Trindade

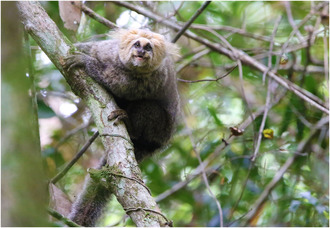

## 1 INTRODUCTION

Non‐human primates play an essential role in their ecosystems, including forest and ecosystem health regeneration (Estrada et al., 2017). However, due to industrialisation and illegal trade of wild animals, they suffer from population decline and face strong loss and fragmentation of their habitat (Estrada et al., 2017). According to a global risk assessment on climate and land use cover for primates worldwide, neotropical primates will be suffering the most, regardless of the scenario (Carvalho et al., 2019). In the Neotropical region, the Atlantic Forest is mainly distributed in Brazil (93%) but also in Paraguay (5.3%) and Argentina (1.7%) (Carlucci et al., 2021). After the Amazonia, the Atlantic Forest is home to the highest primate species diversity (Paglia, 2012) but it is highly fragmented, with a large distance between patches (average of 1440 m in Brazil) (Ribeiro et al., 2009).

Callitrichidae are a family of neotropical arboreal primates including marmosets (*Callithrix* spp.) and tamarins (*Leontopithecus* spp., *Leontocebus* spp. and *Saguinus* spp.) (Byrne et al., 2016; Rylands et al., 2009) that show a typical parapatric distribution pattern (Braz et al., 2019) and strong ecological plasticity (Rodrigues & Martinez, 2014). Previous findings highlight that the genus *Callithrix* can do rapid niche differentiation after species isolation and suggest that some species such as *C. jacchus*, *C. geoffroyi* and to a lower degree, *C. kuhlii* can adapt to warmer environments (Braz et al., 2019). It is important to note, however, that the ongoing deforestation might reduce their ability to adapt to a changing climate (Braz et al., 2019). Indeed, compared to other non‐human primates, arboreal monkey species have limited dispersal capacities (da Silva et al., 2015), which makes them more sensitive to habitat fragmentation and climate change and less likely to find more suitable conditions than non‐human primates that can disperse on the ground (Braz et al., 2019; Rezende et al., 2020).

Among the genus *Callithrix*, most research in the past decades has focused on the common marmosets *C. jacchus* and *C. penicillata* (Hannibal et al., 2019), which are not native to the Atlantic Forest in Southeast Brazil (Braz et al., 2019). However, due to illegal pet trafficking, they became invasive in this region and they represent a serious threat to the endemic, montane species *C. aurita* and *C. flaviceps* (Vale et al., 2020). The common marmoset species seem to be more adaptive, which prejudice the montane species *C. aurita* and *C. flaviceps* (Braz et al., 2019). Despite being the least studied among *Callithrix* species (Hannibal et al., 2019), the montane species *C. aurita* and *C. flaviceps* present the most alarming conservation status (de Melo, Hilário, et al., 2021; de Melo, Port‐Carvalho, et al., 2021).

With a geographical distribution of only 30,815 km^2^ (Malukiewicz, Boere, et al., 2021), *Callithrix flaviceps* has the smallest original geographical range of all South American primates (Hannibal et al., 2019; Mendes & de Melo, 2007; Mittermeier et al., 1980). It was considered Endangered for over 30 years (Hannibal et al., 2019) until it was classified as Critically Endangered since 2020 (de Melo, Hilário, et al., 2021). Its presence on the list of threatened species is due to (1) intensive deforestation across the states of Minas Gerais (MG) and Espírito Santo (ES) (Hirsch et al., 1999), (2) climate change, resulting in dryer and warmer climate within the species range (Carlucci et al., 2021; Townsend, 2008), (3) yellow fever outbreaks, which have highly affected *Callithrix* populations across the states of ES and MG in 2017 (Mares‐Guia et al., 2020; Possamai et al., 2022) and (4) invasive species (especially *C. jacchus* and *C. penicillata)* leading to hybridisation between *Callithrix* species (Braz et al., 2019; Malukiewicz, 2019; Moraes et al., 2019).

*Callithrix flaviceps* usually live in groups of 2–15 individuals (Malukiewicz, Boere, et al., 2021). It prefers secondary forests (Ferrari & Mendes, 1991; Pinto et al., 1993) at altitudes up to 1200 m or more (Ferrari et al., 1996; Moraes et al., 2019). Their upper altitudinal range is limited by a significant decline in precipitation in winter (Ferrari et al., 1996), and low precipitation level is known to negatively impact the abundance of some of the *C. flaviceps* food resources (arthropods) (Corrêa et al., 2000). *Callithrix* species are sensitive to high temperatures (Ferrari, 1988), and only a small increase in temperature would cause strong damage to all marmoset species, including *Callithrix flaviceps* (Braz et al., 2019; Carvalho et al., 2019).

Due to the expected impact of climate change on the distribution of species (Braz et al., 2019; Pearson & Dawson, 2003; Serra‐Diaz & Franklin, 2019), it is urgent to implement conservation areas that will remain suitable under future climate change scenarios, to increase the chances of long‐ term species survival (Mendes & Pereira, 2015). In their study, Rezende et al. (2020) suggest that prioritised areas for the conservation of the endangered black lion tamarin *Leontopithecus chrysopygus* could be both (1) areas with high current climate suitability but low landscape suitability because habitat management and restoration of landscape is possible and (2) areas with low current climate but high landscape suitability which might become highly suitable in light of future climate change scenarios (Rezende et al., 2020). They conclude that niche modelling based on both climatic and landscape variables can provide realistic predictions of habitat suitability and are, therefore, useful tools for defining conservation strategies (Rezende et al., 2020).

Protecting as much as possible of the remaining habitat is the key management response to habitat loss (Townsend, 2008). However, what defines a suitable habitat for *Callithrix flaviceps* is not well defined yet. In this study, Ecological Niche Modelling focuses only on abiotic variables, biotic factors such as species interactions and potential disease outbreaks were not included in the models. We aimed to map the habitat suitability of *C. flaviceps* by modelling the species’ ecological niche (including climate and landscape variables) within its geographical range, and thus, investigate how environmental variables influence habitat suitability. In addition, we used future climate scenarios to identify future likely suitable habitats. Note that, in this study, the following research questions were addressed:

Considering climate and landscape variables, what defines a suitable niche for *Callithrix flaviceps*? How will climate change affect the climatic suitability in the next five decades within the *Callithrix flaviceps* geographical range?
Which area(s) of the current range should be prioritised for the conservation of *Callithrix flaviceps*?
Where should we focus future restoration efforts within the current *Callithrix flaviceps* geographical range?

## 2 METHODS

**FIGURE 1:**
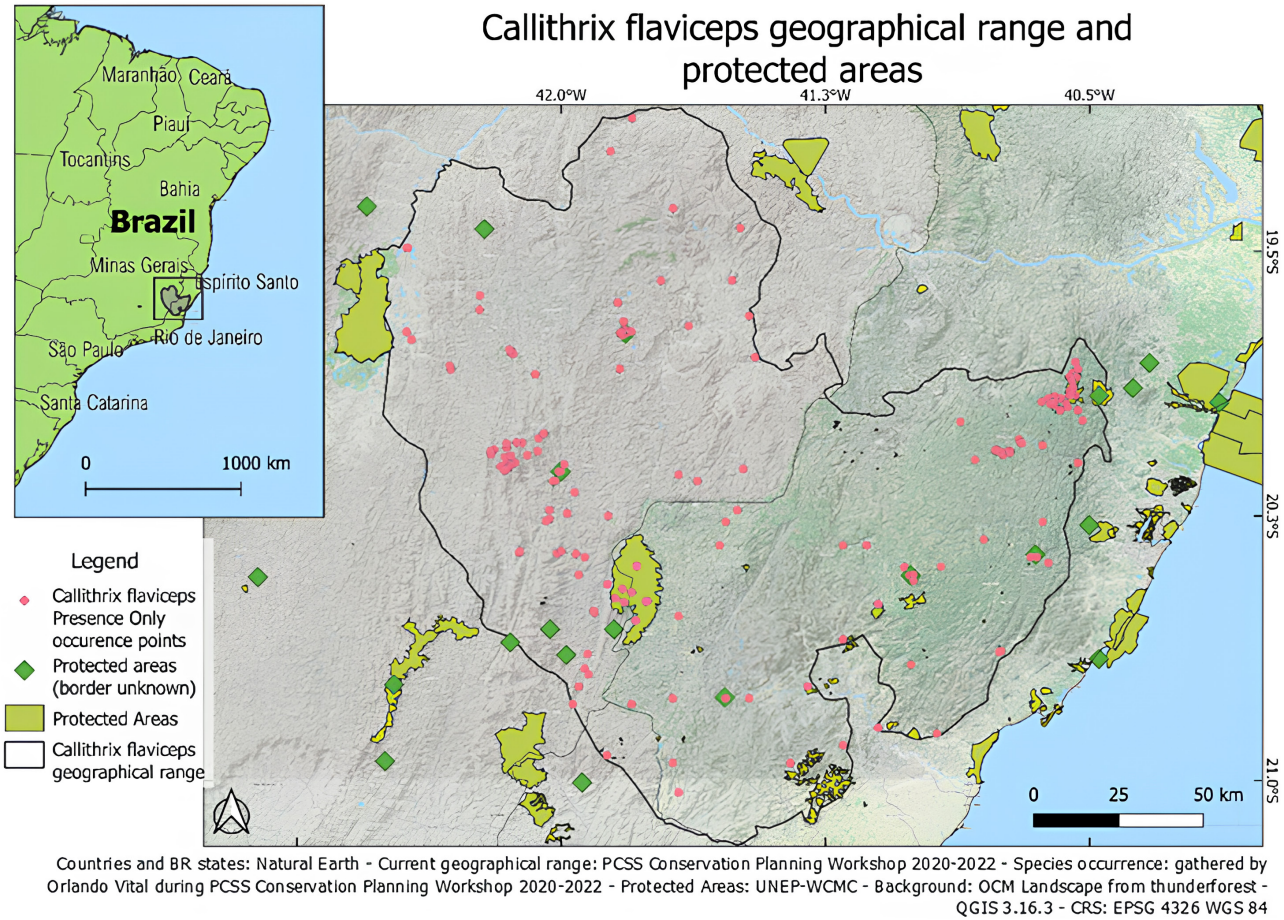
Study area corresponding to the *Callithrix flaviceps* geographical range (2020) and the protected areas (UNEP‐WCMC).

The study area corresponds to the most recent version of the IUCN polygon representing the geographical range *Callithrix flaviceps*, adapted from *PCSS Conservation Planning Workshop 2020–2022 (Orlando Vítor Vital, pers. comm*.; see Figure 1).

### 2.1 Data preparation

Data preparation was done in QGIS (v3.16.3). All variables were set to the EPSG 4326 WGS84 geographical coordinate system.

#### 2.1.1 Occurrence points

We used the dataset ‘Planilha Geral de Dados versão Agosto de 2020’, compiled by Orlando Vítor Vital in 2020 as an effort to gather all available *C. flaviceps* occurrence data. The dataset included 272 presence‐only points from different published and unpublished records for a nearly 60‐year period (1964–2020) across the current geographical range (study area). The data were collected unevenly throughout the period, and the method for data collection was not always known (i.e. method was unavailable for 162 points out of 272). When the method was known, the presence was recorded by observations (sighting) in the wild (*n* = 73), line transect (*n* = 14), citizen science surveys (*n* = 6), museum records (*n* = 5), personal records from different researchers (*n* = 5), camera trap in canopy (*n* = 4) and interviews (*n* = 3) (Figure S1).

### 2.2 Environmental variables

#### 2.2.1 Climate variables

We used current bioclimatic variables from the WorldClim v2.1 database at 30 arc seconds per pixel, referred to as 1‐km spatial resolution (Fick, 2017). For the future climate variables, high‐resolution data (30 arc seconds per pixel) from the Coupled Model Intercomparison Project Phase (CMIP5) from WorldClim *version 1.4* (Humans et al., 2005) were used. Data from 2050 (average of the years 2041–2060) and 2070 (average of the years 2061 and 2080) were selected.

Eight variables were pre‐selected based on the ecology of the Brazilian marmosets. *Callithrix flaviceps* are sensitive to extreme temperature (Braz et al., 2019; Ferrari, 1988), precipitation level is known to impact the abundance of food (arthropods) (Corrêa et al., 2000) and have an impact on the species distribution. Finally, *Callithrix* species seem to prefer seasonal environment (Braz et al., 2019; Corrêa et al., 2000). Based on this information, the following variables were pre‐selected: Annual Mean Temperature (BIO1), Temperature Seasonality (BIO4), Mean Temperature of Wettest Quarter (BIO8), Mean Temperature of Warmest Quarter (BIO10), Mean Temperature of Coldest Quarter (BIO11), Annual Precipitation (BIO12), Precipitation of Wettest Month (BIO13), Precipitation Seasonality (BIO15).

Climatic variables are expected to be correlated; hence, a correlation matrix on current data was run using the R package *PerformanceAnalytics v2.0.4* (Peterson et al., 2020). Based on the correlation coefficients (Figure S2) and study design from previous studies on Brazilian marmosets (Braz et al., 2019), we selected 4 climate variables to include in our modelling.

BIO1 = Annual Mean Temperature (degrees Celsius).
BIO4 = Temperature Seasonality (standard deviation × 100) BIO13 = Precipitation of Wettest Month (unit).
BIO15 = Precipitation Seasonality (Coefficient of Variation).

#### 2.2.2 Climate change scenarios

For simplicity, only one Global Climate Model (GCM) and two Representative Concentration Pathways (RCPs) scenarios were considered in this study. Data from the GCM model HADGEM2‐ ES was coupled with the ‘intermediate’ (RCP4.5) and ‘extreme’ (RCP8.5) RCP scenarios (IPCC, 2022).

#### 2.2.3 Landscape variables

Landscape variables included elevation from a Digital Elevation Model (DEM), at 90 m spatial resolution from EarthEnv (www.earthenv.org) (Robinson et al., 2014), and tree canopy cover (vegetation >5 m tall) for the year 2000 (*treecover2000*), at approximately 30 m spatial resolution from Hansen Global Forest Change *v1.7* (www.earthenginepartners.appspot.com) (Hansen et al., 2013).

### 2.3 Modelling

Ensemble Modelling, in which several different algorithms or training datasets are combined and result in one final prediction (Kotu & Deshpande, 2014), is useful when dealing with uncertainties of extrapolation (Marmion et al., 2009) and to avoid the bias of ‘choosing the best model’ (Guisan et al., 2017). We used the biomod2 package v3.5.1 (Thuiller et al., 2021) in the R software (RStudio Team, 2020) to generate ensemble modes.

#### 2.3.1 Modelling current distribution

As recommended by De Kort et al. (2020), we first considered the climate and landscape variables separately, before combining them in a habitat suitability model (i.e. three maps for climate, landscape and habitat suitability, respectively) (De Kort et al., 2020). Based on this approach, we generated 3 maps of *current* suitability (climate suitability, landscape suitability and overall habitat suitability).

#### 2.3.2 Predicting future distributions under climate change scenarios

Four maps for *future* climate suitability (2 RCP scenarios × 2 years) were created. To help visualise future areas of both high climatic and high landscape suitability, we generated 4 extra maps, each combining one of the 4 future climate scenarios with the (current) landscape variables.

##### Preparing data for modelling

All algorithms available in *biomod2* require presence/absence data, except Maxent (maximum entropy) which uses presence data and a selection of background points. Therefore, 2000 pseudo‐absence points were randomly generated within the study area. As recommended by Guisan et al. (2017), the pseudo‐absence selection was repeated three times to avoid sampling bias, resulting in three different datasets of 2000 pseudo‐absence points each (Guisan et al., 2017).

##### Model calibration to build Ensemble Models

Before ensemble modelling, single models were generated. Seven algorithms were run and evaluated. Algorithms included three statistical regression methods, that is, generalised linear models (GLM) (Guisan et al., 2002), generalised additive models (GAM) (Guisan et al., 2002) and multivariate adaptive regression splines (MARS) (Friedman, 1991) and four machine learning models, that is, random forest (RF) (Breiman, 2001), generalised boosting models (GBM/BRT) (Elith et al., 2008), classification tree analysis (CTA) (Vayssières et al., 2000) and maximum entropy (MAXENT) (Phillips et al., 2006).

In GLMs, we included the quadratic terms and two‐way interactions for all variables. The number of tree limits in RFs, and GBMs was kept as their default in package biomod2 (500 and 2500, respectively; see the package's documentation for details). MAXENT's maximum iterations were set to 1000.

Independent data were not available to evaluate the models. We therefore evaluated the predictive performance of the models using a k‐fold cross‐validation procedure. We used the relative operative characteristic (ROC) and the true skill statistics (TSS) metrics to evaluate the model performance.

#### 2.3.3 Variable importance

The variable importance was checked for each of the models. To make results more readable and to get the variable importance per algorithm, results were averaged among the cross‐validation procedures (runs) and the pseudo‐absence selections. We set the number of permutations for variable importance to 7 (Thuiller et al., 2021).

#### 2.3.4 Response curves

Once the variable importance was known, we investigated how each of them was related to the species' probability of presence. We used the evaluation strip procedure proposed by Elith et al. (2006) from the R package *biomod2* (Thuiller et al., 2021) to plot response curves for each variable, for each algorithm. Note that each response curve is modelled considering all other variables included in the model being held constant (Guisan et al., 2017).

#### 2.3.5 Ensemble modelling

We used a TSS threshold of 0.7 to build the final ensemble model, meaning that only models with a TSS ≥0.7 were kept for the final ensemble models. Final ensemble models were then evaluated using the same metrics as the individual models (ROC and TSS).

To have both predictions and confidence intervals around these predictions, five ‘ensembling’ options were considered: the *mean*, giving the mean probabilities of occurrence across predictions; the *weighted mean* (wmean), estimating the weighted sum of probabilities; the *committee averaging* (ca), giving both a prediction and a measure of uncertainty and the *confidence interval* (95%) (ci), showing 2 estimations (the lower one: ci inf and the upper one: ci sup) of the confidence interval around the *mean* probability.

Variable importance was also generated for the Ensemble Models.

#### 2.3.6 Current and future projections and Ensemble Forecasting

The ensemble models for current distribution built under the weighted mean rule were used to generate the projections on *current* and *future* spatial distribution. We ran this step for climate suitability in the years 2050 and 2070, each based on current projections and trained models (from the *Ensemble modelling* step) with two climate change scenarios (RCP4.5 & RCP8.5).

## 3 RESULTS

### 3.1 Overall habitat suitability

#### 3.1.1 Individual models

We created a total of 63 models for the overall habitat suitability, combining both climate and landscape variables (3 pseudo‐absence selections × 3 cross‐validation procedures (runs) × 7 algorithms).

As for both climatic and landscape suitability modelling, the machine learning methods (the random forest RF and to a lesser extent the Generalised Boosting Models GBM and Classification Tree Analysis CTA) tended to perform better. In this case, RF stood out as the best model based on the TSS and ROC (Figure S3).

#### 3.1.2 Response curves

Suitability clearly dropped at an Annual Mean Temperature (BIO1) of 20°C (17/18°C in the climate‐only models). At an Annual Mean Temperature of 20°C or higher, all algorithms except random forest models (RF) showed the low relative probability of presence (Figures S4–S10).

All algorithms except GLMs and CTAs predicted a higher probability of species occurrence when Precipitation Seasonality (BIO15) was below 55% or above 70%.

According to GLM, MARS and MAXENT, when combining all environmental variables, elevation above 1000 m seemed to be unsuitable for *Callithrix flaviceps*. Yet, all algorithms predicted a positive relationship between elevation and probability of presence when including only the landscape variables. The machine learning methods GBM and RF seemed more consistent and showed similar pattern as they did in landscape‐only modelling (response curves from climate‐only and landscape‐only modelling are available in Appendix S1 Part 2 and 3 respectively).

#### 3.1.3 Ensemble modelling

Based on the TSS threshold, 5 individual models were kept for EM. As explained above, five ensemble models were created: ‘PA1_RUN2_RF’, ‘PA1_RUN3_RF’, ‘PA3_RUN1_RF’ ‘PA3_RUN2_RF’, ‘PA3_RUN3_RF’.

Table S2 of supplementary materials shows the five ensemble model scores. We discuss only the weighted mean (wmean) as it consistently performed better than the other ensembling methods.

#### 3.1.4 Variable importance

Precipitation Seasonality (BIO15) and Tree cover were the most important variables in climate‐based modelling and landscape‐based modelling, respectively (supplementary materials, part 3, Figures S25 and S26). Interestingly, statistical methods (GLM, GAM and MARS) gave varying results while modelling overall habitat suitability: In landscape‐based modelling, statistical methods (GLM, GAM and MARS) considered tree cover to be more important than elevation (DEM). In the habitat modelling however, (when combined with climate variables), elevation was considered more important than tree cover by all statistical methods (GLM, GAM, MARS).

**FIGURE 2:**
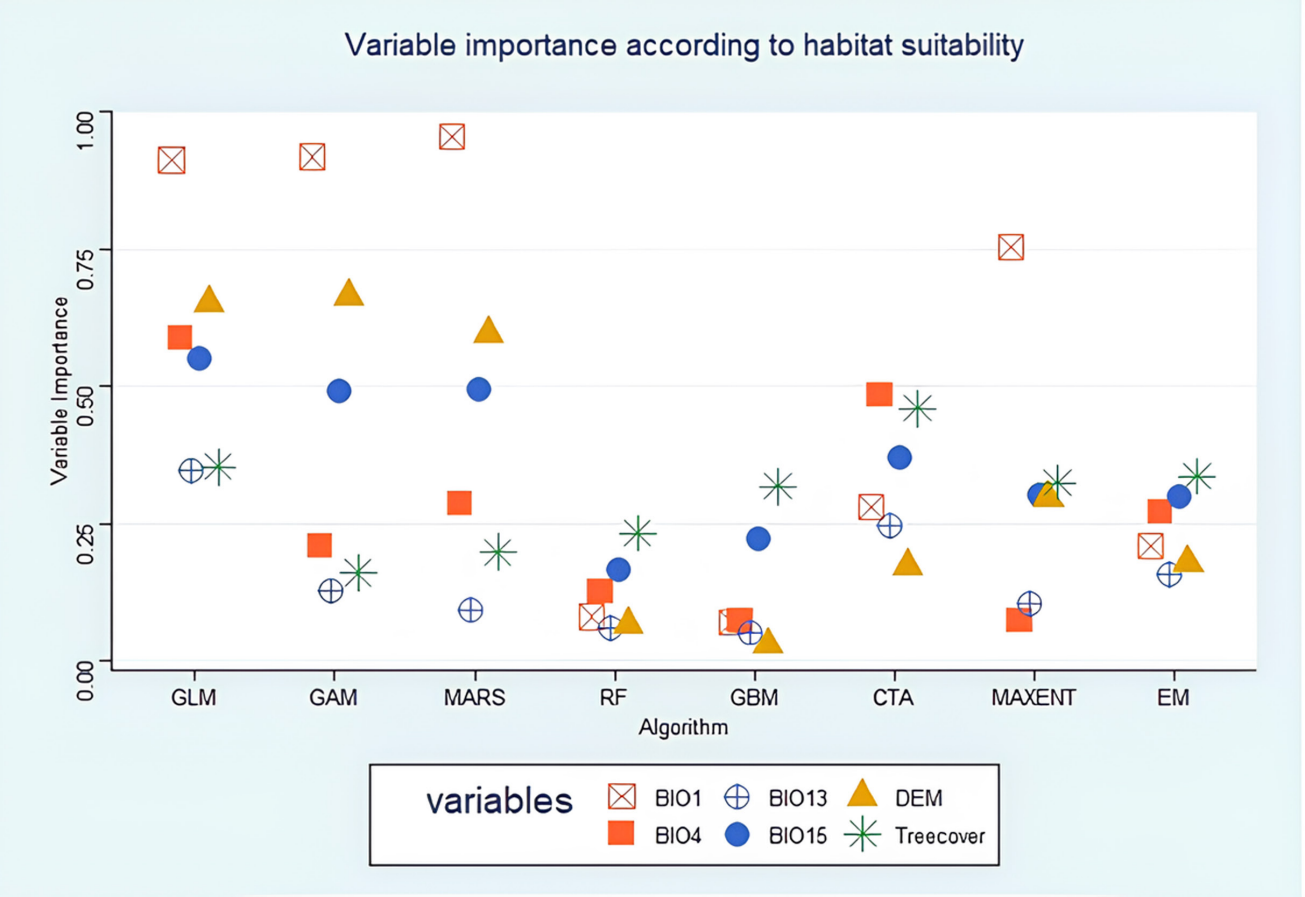
Variable importance for each algorithm and ensemble model for predicting the current habitat suitability of *Callithrix flaviceps* using both climatic and landscape variables. Values closer to 1 are considered more important for the prediction of distribution. Variables were Annual mean Temperature (BIO1), Temperature Seasonality (BIO4), Precipitation Wettest Month (BIO13), Precipitation Seasonality (BIO15), Digital Elevation Model (DEM), Tree Canopy Cover (Treecover). Algorithms were GLM: Generalised Linear Models, GAM: Generalised Additive Models, MARS: Multivariate Adaptive Regression Splines, RF: Random Forest, GBM: Generalised Boosting Models, CTA: Classification Tree Analysis, MAXENT: Maximum Entropy, EM: Ensemble Models.

Similarly, in climate‐based modelling, all algorithms agreed on Precipitation Seasonality (BIO15) being the most important climate variable. However, when combined with landscape variables (overall habitat suitability), statistical methods (GLMs, GAMs, MARSs) and MAXENT considered Annual Mean Temperature (BIO1) to be the most important variable (Figure 2).

Note however that statistical methods and MAXENT had the lowest scores (ROC? TSS? Ref to where this result can be found). The machine learning methods RF and GBM seemed more consistent. They considered Precipitation Seasonality (BIO15) and tree cover the two most important variables (Figure 2), which is in accordance with the climate‐based and landscape‐based models done separately (supplementary materials, part 3, Figures S25 and S26).

Based on EM, the most important variable to predict the species' distribution was tree cover, followed by Precipitation Seasonality (BIO15) and Temperature Seasonality (BIO4). Elevation (DEM) and Precipitation of Wettest Month (BIO13) were considered the least important variables.

#### 3.1.5 Current and future projections and Ensemble forecasting

The current projection for overall habitat suitability is shown in Figure S27 of supplementary materials.

Combining climate and landscape variables, the Caparaó National Park is still considered a suitable area as well as the Biological Reserve Augusto Ruschi. However, the Northeast part of the geographical range and Goiapaba‐Açu area (in Espírito Santo State) are now considered less suitable.

**FIGURE 3:**
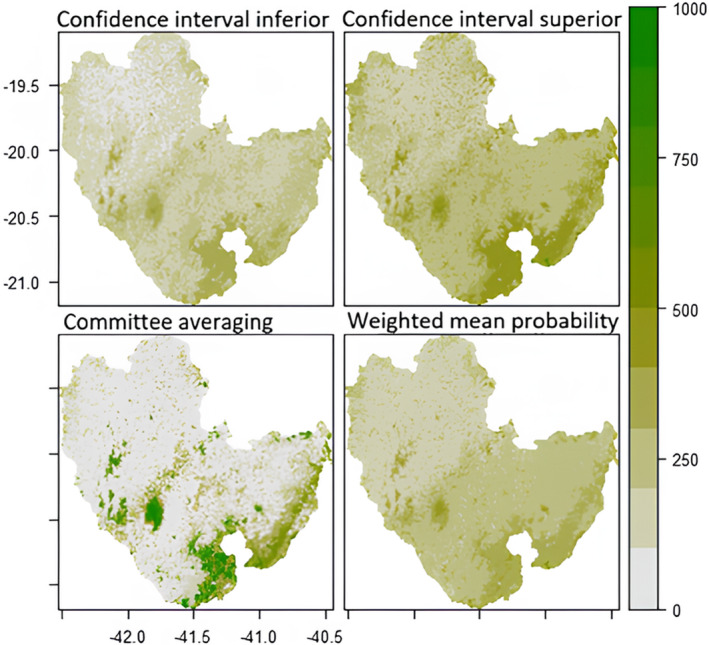
Future (2050) prediction of overall habitat suitability for *Callithrix flaviceps* under climate scenario RCP45. Each map represents a different Ensemble Model. The two top maps show two levels of confidence interval around the mean probability. The committee averaging shows the consistency between models: dark green indicates that all models agreed to predict presence, whereas grey indicates that all models agreed to predict absence of the species; yellow/light green shows inconsistencies between model predictions. The weighted mean probability represents the actual ensemble prediction, with dark green representing highly suitable areas.

**FIGURE 4:**
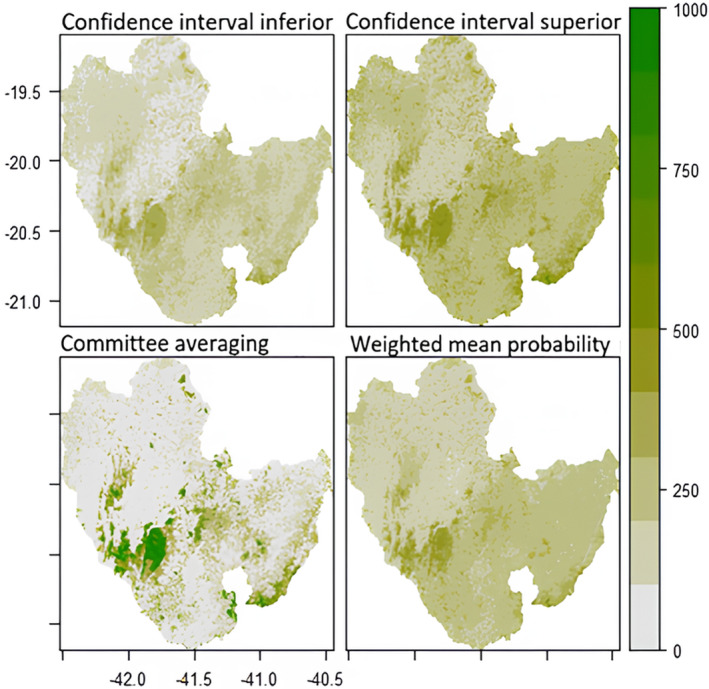
Future (2070) prediction of overall habitat suitability for *Callithrix flaviceps* under climate scenario RCP45. Each map represents a different Ensemble Model. The two tops maps show two levels of confidence interval around the mean probability. The committee averaging shows the consistency between models: dark green indicates that all models agreed to predict presence, whereas grey indicates that all models agreed to predict absence of the species; yellow/light green shows inconsistencies between model predictions. The weighted mean probability represents the actual ensemble prediction, with dark green representing highly suitable areas.

**FIGURE 5:**
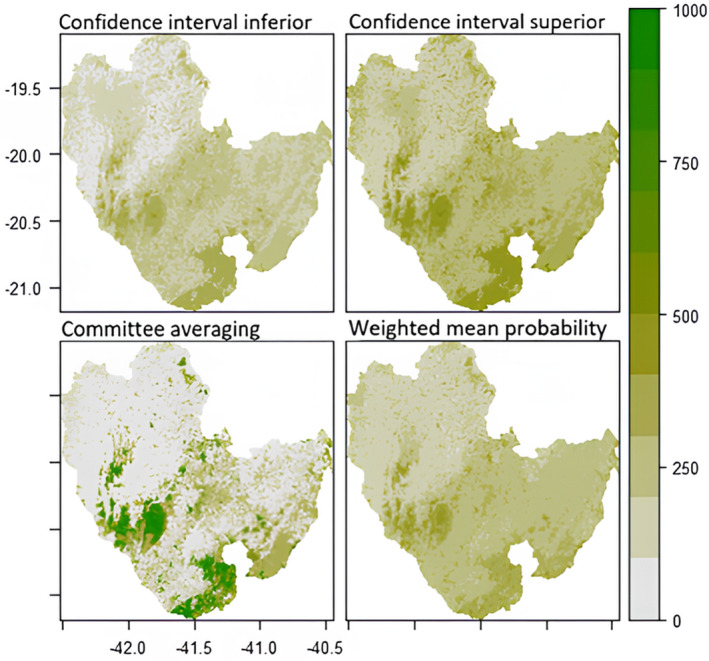
Future (2050) prediction of overall habitat suitability for *Callithrix flaviceps* under climate scenario RCP85. Each map represents a different Ensemble Model. The two tops maps show two levels of confidence interval around the mean probability. The committee averaging shows the consistency between models: dark green indicates that all models agreed to predict presence, whereas grey indicates that all models agreed to predict absence of the species; yellow/light green shows inconsistencies between model predictions. The weighted mean probability represents the actual ensemble prediction, with dark green representing highly suitable areas.

**FIGURE 6:**
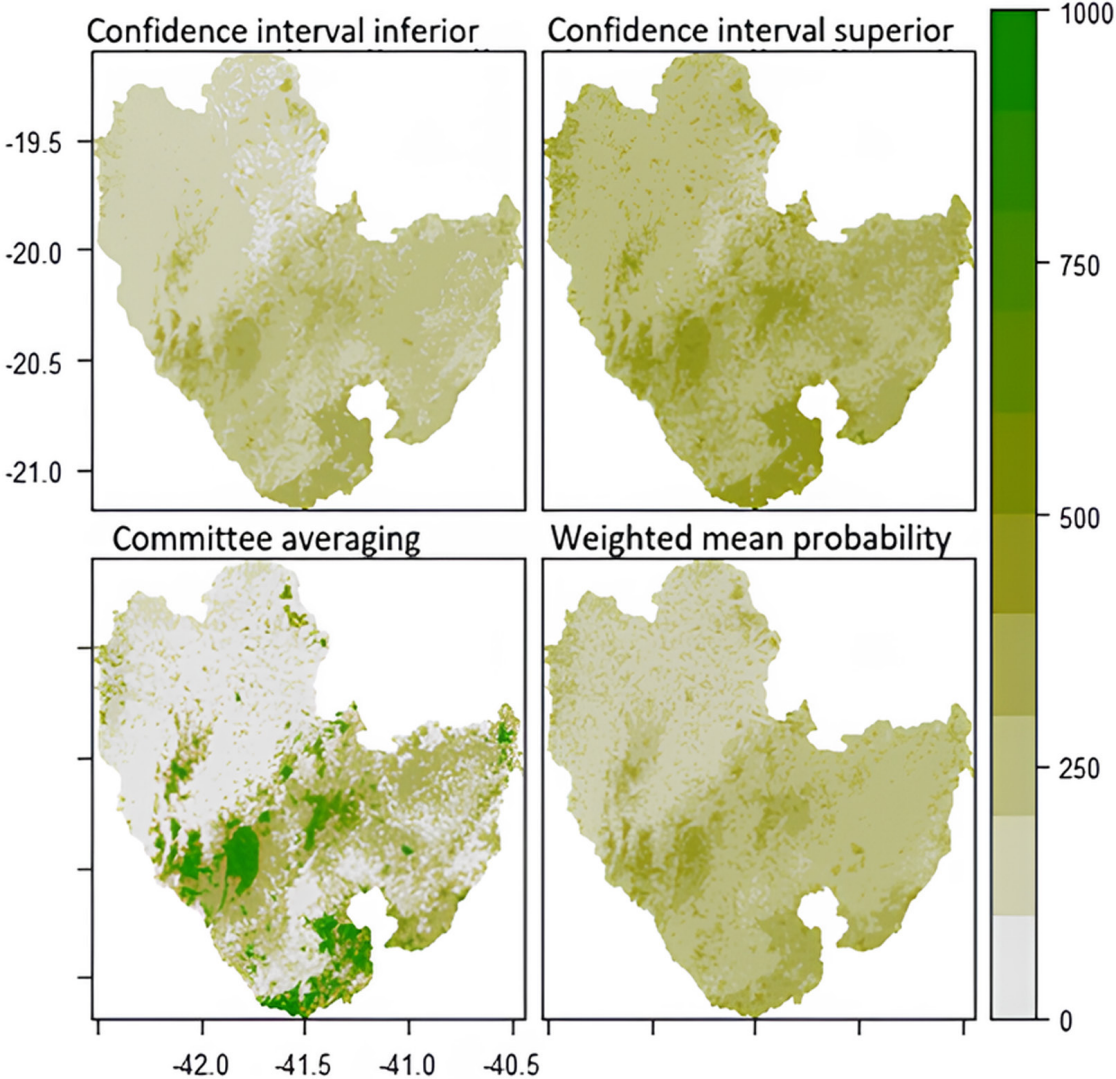
Future (2070) prediction of overall habitat suitability for *Callithrix flaviceps* under climate scenario RCP85. Each map represents a different Ensemble Model. The two tops maps show two levels of confidence interval around the mean probability. The committee averaging shows the consistency between models: dark green indicates that all models agreed to predict presence, whereas grey indicates that all models agreed to predict absence of the species; yellow/light green shows inconsistencies between model predictions. The weighted mean probability represents the actual ensemble prediction, with dark green representing highly suitable areas.

The following maps (Figures 3, 4, 5, 6) were created to visualise the projected impact of the different climate scenarios on overall habitat suitability (climate + landscape). We used the *current* landscape and predicted future climate layers to create the projections maps for habitat suitability. In each figure, the weighted mean of the probability of occurrence from the ensemble model is shown on the bottom right map. Inconsistency between models is shown on the committee averaging map (bottom left), as well as the two confidence levels around the mean of probability (top maps).

Regardless of the scenario, the Caparaó National Park (protected area between Minas Gerais (MG) and Espírito Santo (ES) States) and the southeast of the range (around Serra das Torres protected area in ES) are projected to be highly suitable and at high consistency between models in 2050. However, committee averaging for the future predictions shows inconsistency between models, for the year 2070 for the extreme scenario (RCP8.5) (light green/yellow colours).

All areas that are white in the bottom left maps (committee averaging) show high certainty of unsuitable areas. Most areas located in Espírito Santo show high inconsistency (for all projections).

None of the areas showed high overall habitat suitability within this scenario (all areas <60% suitability, however, some hotspots are found).

**FIGURE 7:**
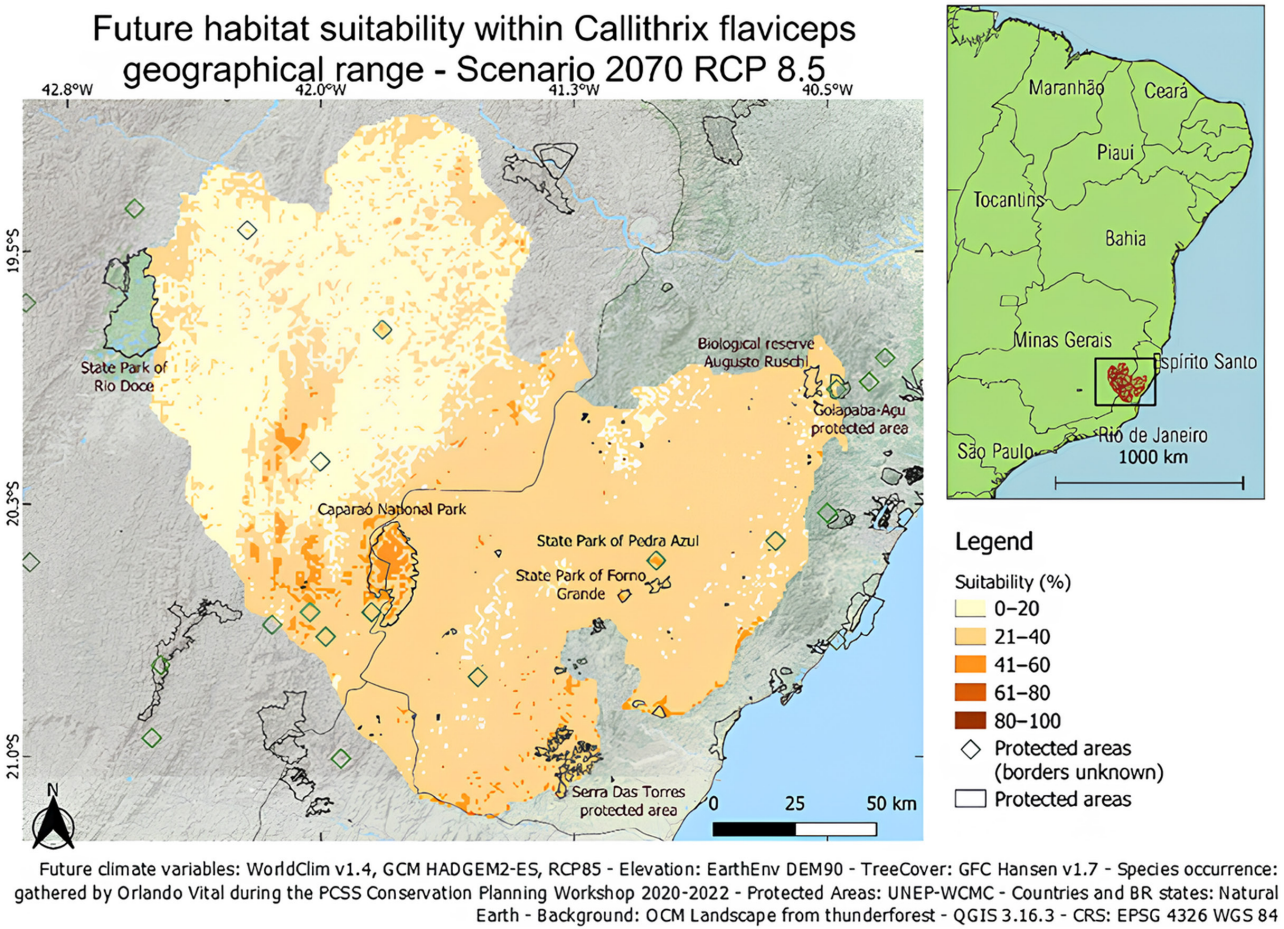
Projected overall habitat suitability for *Callithrix flaviceps*, combining both climate and landscape variables for the year 2070 considering the extreme climate change scenario (RCP8.5).

Considering the pressure from anthropogenic activities, the extreme RCP8.5 scenario is the most likely to happen in South America (Cuyckens et al., 2016). Hence, habitat suitability projection for 2070 considering the extreme climate scenario (RCP8.5) and protected areas already in place, is shown in Figure 7.

The Caparaó National Park represents a moderately suitable area with high consistency in all scenarios. Among all four scenarios (2 RCP × 2 years), models predict suitability at 30%–60% depending on the scenario, considering both climate‐only and habitat suitability modelling. Climate‐only and overall habitat suitability predictions show the same pattern. Based on these results, this already protected area should be maintained and is likely to be a key area for the persistence of *Callithrix flaviceps*.

## 4 DISCUSSION

The aim of this study was to determine what defines a suitable ecological niche for *Callithrix flaviceps* and to map likely suitable areas under future climate change scenarios. To do so and as recommended by Rezende et al. (2020) climate niche‐based, and landscape niche‐based modelling were run first separately, then together (Rezende et al., 2020). Climate change scenarios from two Representative Concentration Pathways (RCP4.5 and RCP8.5) for the years 2050 and 2070 were generated to determine the future climatic suitability of the focal species. Based on these results, and with the caution of the limitations outlined below, we propose recommendations for conservation and restoration efforts.

In our dataset, bias was introduced in the occurrence dataset in different ways: one being the opportunistic data collection in some cases (citizen science), which can introduce a bias toward observations in more accessible areas whereas the species might be present in areas not registered simply because nobody was actively searching for them. Another source of bias could come from the several decade time span of the dataset and the uneven spatial distribution of the occurrence points. The species might already be gone from areas where they were found in the 1960s. Moreover, the Atlantic Forest being highly fragmented, some areas where the species currently exists, may not be a real source for dispersal to adjacent areas that are assumed to be suitable in the future.

In their study on geographic distribution of all *Callithrix* species in southeast Brazil, Braz et al. (2019) concluded that, unlike most *Callithrix* species, parapatry of *Callithrix flaviceps* was mostly maintained by biotic factors (i.e. interspecific interactions with *C. aurita and C. penicillata*) (Braz et al., 2019). Furthermore, the effect of the invasive species *C. penicillata* and *C. jacchus* and their range expansion leading to hybridisation are not to be negligible (Malukiewicz, Boere, et al., 2021). Consequently, future work including species interactions, direct (leading to hybridisation) and indirect (leading to competition), especially with *C. aurita* and the invasive marmosets *C. penicillata* and *C. jacchus* are needed to get a better picture of the realised niche of *C. flaviceps*.

Using ensemble modelling in this study has shown the importance of including both climate and landscape variables to model habitat suitability. Indeed, four climate variables and two landscape variables were included in the models. Interpretation from statistical methods (GLM, GAM, MARS) and MAXENT was different when modelling climate and landscape variables separately (climatic suitability and landscape suitability) versus together (overall habitat suitability). On the other hand, the machine learning methods Random Forest (RF) and Generalised Boosting Method (GBM) performed better and were more consistent, which was supported by Elith et al. (2006) stating that boosting and bagging methods usually have higher predictive performance (Elith et al., 2006).

Landscape‐based niche models had lower prediction scores than climate‐based models and overall (landscape + climate) habitat models, which can be explained by the low number of variables included. In this study, however, we aimed to predict the niche of *Callithrix flaviceps*, and its potential changes in distribution over the next decades. For these specific aims, Rezende et al. (2020) stated that climate variables are more relevant than landscape variables (Rezende et al., 2020).

### 4.1 Relevant factors for defining the niche

Precipitation level is directly related to food availability, less precipitation means less arthropods (Corrêa et al., 2000). This could explain why *C. flaviceps* seem to prefer higher precipitation seasonality (70%). However, the wide ecological plasticity of *Callithrix* spp. has been investigated (Ferrari et al., 1996), and it is understood that they can easily change their diet according to the change in environment (Rodrigues & Martinez, 2014). For example, *Callithrix flaviceps* is known to switch from a gummivorous/insectivorous diet to feeding more on fruits when these latter were more abundant in the Caratinga Biological Station (Ferrari et al., 1996).

The current and previous studies have provided knowledge about the fundamental niche of *Callithrix flaviceps*, but it is challenging to know the realised niche because of constraints from biotic factors such as resource competition, disease outbreaks and hybridisation. Hence, to have a broader picture and more accurate projections of the realised niche, studies investigating species interactions relative to landscape and climatic factors are needed, including interactions with *C. aurita*, but also with the common and invasive marmosets *C. penicillata* and *C. jacchus*, as well as metrics such as landscape variables to predict constraints on *C. flaviceps* movement and dispersal capacity (Rezende et al., 2020). In addition, as proposed by Hannibal et al. (2019), agricultural land and urban areas should be included in the next conservation strategies to better consider habitat fragmentation (Hannibal et al., 2019).

### 4.2 Current projections

In this study, the climatic processes precipitation seasonality and temperature seasonality seemed the most important to predict the species distribution. These results align with previous studies highlighting the importance of seasonality for *Callithrix* spp. (Vale et al., 2020), and with the tendency for montane species such as *C. aurita* and *C. flaviceps* to occupy colder environments than other *Callithrix* species (Braz et al., 2019) (Figure S25).

Regarding the landscape variables, tree cover played a major role in predicting distribution and was considered more decisive than elevation. It is known however, that elevation is more likely to have an indirect effect on species distribution because altitude influences temperature (Araújo et al., 2005). Hence, for modelling, it is better to use variables that have direct effects on distribution only (Araújo et al., 2005).

Most hotspots of current high suitability, as predicted by both climate‐ and landscape‐based models, correspond to the already existing protected areas (Figures S22 and S23 of Supplementary materials, see Figure S18 for protected area names). This is also the case for many of the clusters of presence‐only points that the analyses were based on. For example, the Caparaó National Park between the states of MG and ES, the Biological Reserve Augusto Ruschi, the Goiapaba‐Açú area (including both the environmental protection area and municipal park), the RPPN Águia Branca and the State parks of Forno Grande and Pedra Azul located in Espírito Santo are hotspots of greatest habitat suitability.

### 4.3 Projections under future climate change scenarios

Projections showed fair scores (TSS > 0.4 and ROC >0.7), nevertheless, results must be interpreted with caution. Committee averaging maps account for variation between models.

Regardless of the scenario, projections for future suitability between climate‐only and overall suitability showed similar results. However, the committee averaging map showed more inconsistency for the overall habitat suitability model (Figures 3, 4, 5, 6) than for the climate‐only model (Figurse S28–S31), that is, being sensitive to small samples, the models had more difficulties agreeing whether the species would be present or absent in specific areas. In addition, committee averaging from overall habitat suitability modelling predicted more areas of absence of the species. This points out that landscape variables had a big impact and confirms that tree cover should be maintained/restored. As an arboreal species, it is evident that tree cover plays a major role in *Callithrix flaviceps* distribution.

In the climate‐only modelling, Espírito Santo seemed highly suitable and with high consistency between models to predict presence. In the overall habitat modelling, most areas of the geographical range showed inconsistency between models. However, hotspots such as the Caparaó National Park, the southeastern region of the geographical range (around Serra das Torres) and the State Park of Pedra Azul and Forno Grande were predicted as the presence with high consistency between models.

In their study on the effects of climate change on all six *Callithrix* species in eastern Brazil, Braz et al. (2019) predicted that by 2050, under the RCP8.5 scenario, the east side of Espírito Santo would be the only suitable area remaining from the current geographical range. Indeed, they predicted that the eastern side of the geographical range would be the only remaining suitable area. In the current study, the same area showed high inconsistencies between models but was projected as poorly suitable in the future. Their findings predicted that all *Callithrix* species would maintain their current distribution areas, except for *C. flaviceps*, for which the area would decrease by 95% (1505 km^2^) (Braz et al., 2019). Overall, present results show more suitability within the state of Espírito Santo than in Minas Gerais and Rio de Janeiro. Accordingly, Pinto et al. (2009) predicted that hotspots for the genus *Callithrix* correspond to the forest of Espírito Santo (Pinto et al, 2009).

In contrast with Braz et al. (2019), the recent study of Amaral et al. (2023) reporting the resilience of the genus *Callithrix* due to climatic changes in the Atlantic Forest and the evolving environmental policies in Brazil, shows that all six species except *Callithrix jacchus* will lose 30 to 70% of potentially suitable habitats between 2040 and 2060 (Amaral et al., 2023). In addition, their study highlights that a higher competition for resources is expected in Southeast Brazil because of the occurrence of C*. penicillata* and *C. jacchus* (despite the general reduction of suitable areas for these two species in this region) (Amaral et al., 2023).

## 5 RECOMMENDATIONS FOR CONSERVATION

The prediction of future habitat suitability for *Callithrix flaviceps* resulting from the current study is highly uncertain in the Northeast region of the current geographical range (around Augusto Ruschii and Goiapaba‐Açu protected areas), and further studies would be recommended. Consequently, it does not seem to be a prioritised area for conservation of *Callithrix flaviceps*, even though this is one of the areas with the largest number of occurrence points. Note however, that most occurrence points collected in this region are fairly old (1960s–1980s) except inside the protected area of Augusto Ruschi, for which occurrences were more recent (2000s, 2010s).

Restoration of Serra Das Torres should be considered since this protected area was more suitable considering the climate‐only variables regardless of the scenario, and consistently indicating presence from committee averaging from both habitat and climate‐only modelling.

Suitability hotspots did not seem to follow the terrain elevation pattern except for the year 2050 considering RCP8.5. Indeed, except for Caparaó, the areas of highest projected current and future suitability seemed to match with the lowest altitudes. Interestingly, around the Fazenda Macedônia east side of Minas Gerais, elevation is low (<400/500 m a.s.l.) and on the southeastern part of the current range around Serra das Torres, where projected suitability is good, elevation is also low, especially compared to Caparaó National Park (highest point at 2800 m a.s.l.).

da Silva et al. (2015), highlighted that being a rare species, the geographical distribution of *Callithrix flaviceps* may be underestimated (da Silva et al., 2015). A population of *C. flaviceps* has recently (2023) been observed in Sete Salões State Park, municipality of Conselheiro Pena, MG (*personal communication*, Samuel Lucas Brasileiro, Centro de Conservação dos Saguis‐da‐Serra, Viçosa, MG). This is promising and suggests potentially suitable areas outside the current geographical range.

Genetic studies seem a great way for long‐term monitoring and conservation of rare species. However, to our knowledge, no genetic data is available for *Callithrix flaviceps*. As suggested by Malukiewicz, Boere, et al. (2021), *Callithrix flaviceps* would highly benefit from the genomic skimming method and low‐cost desktop sequencing to rapidly increase its genomic resources, and thus, obtain deeper coverage of genomes (including mitogenomes) (Malukiewicz, Cartwright, et al., 2021). Monitoring the genetic variation within the population and inbreeding levels, to start a healthy population in captivity but also to verify the hybridisation level of the individuals, seem necessary. Recently, Malukiewicz, Boere, et al. (2021) discovered that an individual with a *C. aurita* phenotype was in reality a cryptic hybrid with a *C. penicillata* mitogenome lineage. The hybrid population might therefore be underestimated (Malukiewicz, Cartwright, et al., 2021).

Sadly, while hybridisation is considered one of the threats to the buffy‐headed marmoset, we might have to consider the hybrid population (i.e. between the common/invasive marmosets *C. penicillata* and *C. jacchus* with the endemic marmosets *C. aurita* and *C. flaviceps*) as one of the more realistic scenarios for the rescue of these species in the near future.

## 6 MODELLING LIMITATIONS AND POTENTIAL IMPROVEMENTS

The authors would like to address some potentials for improvements for future work on environmental niche modelling.

First, the use of the variance inflation factor (VIF) can help identify multi‐correlated variables (Zuur et al., 2010). Next, as stated earlier, part of the occurrence data in this present study was from citizen science, with a lack of consistency in the data collection. However, it is crucial to note that citizen science can give solid input for improving modelling and prediction accuracy for species for which more systematic data are lacking. If biases are well accounted for, using citizen science is better than using random pseudo‐absence (Matutini et al., 2021; Milanesi et al., 2020).

Potential improvements in relation to the predictors could be considered. In this paper, the climatic variables we used have helped to get a better understanding of the *C. flaviceps'* niche, however, for further study, we suggest the use of other climatic variables, such as ‘precipitation of driest month (BIO14)’. Several papers stress the issue of using adequate variables, also including non‐climatic variables such as (Della Rocca & Milanesi, 2020; Howard et al., 2023; Milanesi et al., 2022; Milanesi, Breiner, et al., 2017; Milanesi, Holderegger, et al., 2017; Thuiller et al., 2018; Titeux et al., 2016).

In our models, we did not account for spatial autocorrelation and our dataset did not allow us to account for any bias in sampling effort, which may have led to biases in our predictions (Doser et al., 2022). Site‐occupancy models can be a great way to deal with imperfect species detection (Della Rocca & Milanesi, 2020; Doser et al., 2022; Kellner et al., 2023), although these models were not appropriate in our present study due to the lack of repeated visits at sampling sites and the limited number of species locations. Lastly, we would like to mention the recently developed method R‐INLA SPDE, a Bayesian approach accounting for spatial dependencies in species locations (Moraga et al., 2021) which seems promising and allows for more flexibility in the modelling process (Della Rocca & Milanesi, 2022a, 2022b).

Despite the lack of consistency in the presence records, our results have highlighted some important variables affecting the *Callithrix flaviceps* distribution. We found that most hotspots of overall habitat suitability were matching with already existing protected areas, which is promising. However, the Atlantic Forest being a fragmented environment, these potentially suitable areas will be lost if connectivity is not improved. Moreover, species interactions and hybridisation with the common marmosets are making the situation more challenging. Further work is needed to confront present findings and to investigate the realised niche of *Callithrix flaviceps* more in‐depth. Efforts for conservation are still desperately needed.

## AUTHOR CONTRIBUTIONS

**Léa Bataillard:** Conceptualization (lead); data curation (equal); formal analysis (lead); funding acquisition (supporting); investigation (equal); methodology (lead); resources (lead); software (equal); validation (lead); visualization (lead); writing – original draft (lead); writing – review and editing (lead). **Ane Eriksen:** Conceptualization (lead); funding acquisition (equal); investigation (equal); methodology (lead); resources (equal); supervision (lead); validation (lead); visualization (equal); writing – original draft (supporting); writing – review and editing (lead). **Fabiano R. de Melo:** Conceptualization (lead); data curation (supporting); methodology (equal); project administration (lead); resources (lead); supervision (lead); validation (lead); writing – review and editing (equal). **Olivier Devineau:** Conceptualization (equal); data curation (supporting); formal analysis (equal); funding acquisition (equal); methodology (lead); resources (lead); supervision (equal); validation (equal); visualization (equal); writing – review and editing (equal). **Adriana Pereira Milagres:** Conceptualization (equal); formal analysis (equal); methodology (lead); resources (equal); writing – review and editing (equal). **Orlando Vítor Vital:** Conceptualization (equal); data curation (lead); methodology (equal); project administration (equal); resources (equal); writing – review and editing (supporting).

## CONFLICT OF INTEREST STATEMENT

The authors declare no conflict of interests.

### OPEN RESEARCH BADGES

This article has earned an Open Data badge for making publicly available the digitally‐shareable data necessary to reproduce the reported results. The data is available at https://datadryad.org/stash/share/4wkvcHfcB3nYSFrUDtN9i‐KPXROHwDXnt5ayc1OmYYQ.

## Supporting information


Appendix S1.

## Data Availability

All the data and R scripts to analyse them will be available on Dryad (DOI: https://datadryad.org/stash/share/4wkvcHfcB3nYSFrUDtN9i‐KPXROHwDXnt5ayc1OmYYQ).
