# Supplementary material for: Using ecological niche modelling to prioritise areas for conservation of the critically endangered Buffy‐Headed marmoset (Callithrix flaviceps)
Source: Ecol Evol. 2024 Apr 5;14(4):e11203. doi: 10.1002/ece3.11203 (PMC10995821; doi:10.1002/ece3.11203)
Supplement: Supplementary file 1 — Appendix S1. [file ECE3-14-e11203-s001.docx]

**Supplementary materials:**

*Table 1: Method of data collection for the C. flaviceps occurrence points. Data were gathered by Orlando Vital as part of the PPSS Workshop, 2020.*

|  | Sighting | Line transect | Citizen science | Museum | Personal records | Camera trap | Interview | NA |
| --- | --- | --- | --- | --- | --- | --- | --- | --- |
| 1964 | 1 |  |  |  |  |  |  | 12 |
| 1969 |  |  |  | 1 |  |  |  |  |
| 1977 |  |  |  | 1 |  |  |  |  |
| 1980 | 1 |  |  |  |  |  |  | 2 |
| 1982 |  |  |  |  |  |  |  | 1 |
| 1984 |  |  |  |  |  |  |  | 3 |
| 1986 | 3 |  | 1 |  |  |  |  |  |
| 1987 | 1 |  |  |  |  |  |  |  |
| 1988 | 1 |  |  |  |  |  |  | 3 |
| 1991 | 8 | 1 |  |  |  |  |  | 30 |
| 1992 | 1 |  |  |  |  |  |  |  |
| 1993 | 11 | 1 |  |  |  |  | 2 | 27 |
| 1995 | 1 |  |  | 1 |  |  |  |  |
| 1996 | 5 |  |  |  |  |  |  |  |
| 1997 | 1 |  |  | 1 |  |  |  | 14 |
| 1999 | 4 |  |  |  |  |  |  | 2 |
| 2000 | 1 |  |  | 1 |  |  |  | 8 |
| 2001 |  |  |  |  |  |  |  | 2 |
| 2002 |  |  |  |  |  |  |  | 1 |
| 2003 | 3 | 11 |  |  |  |  |  | 1 |
| 2005 | 14 |  |  |  |  |  |  |  |
| 2006 | 1 |  | 1 |  |  |  | 1 |  |
| 2009 |  |  |  |  |  |  |  | 2 |
| 2010 |  |  |  |  |  |  |  | 1 |
| 2011 |  |  |  |  |  |  |  | 1 |
| 2012 |  | 1 |  |  |  |  |  | 7 |
| 2013 |  |  |  |  |  |  |  | 12 |
| 2014 |  |  |  |  |  |  |  | 5 |
| 2015 | 3 |  |  |  |  |  |  | 18 |
| 2016 | 1 |  | 1 |  |  |  |  |  |
| 2017 | 1 |  |  |  |  | 3 |  |  |
| 2018 | 4 |  | 3 |  | 5 | 1 |  | 9 |
| 2019 | 6 |  |  |  |  |  |  |  |
| 2020 | 1 |  |  |  |  |  |  | 1 |
| Grand Total | **73** | **14** | **6** | **5** | **5** | **4** | **3** | **162** |

*
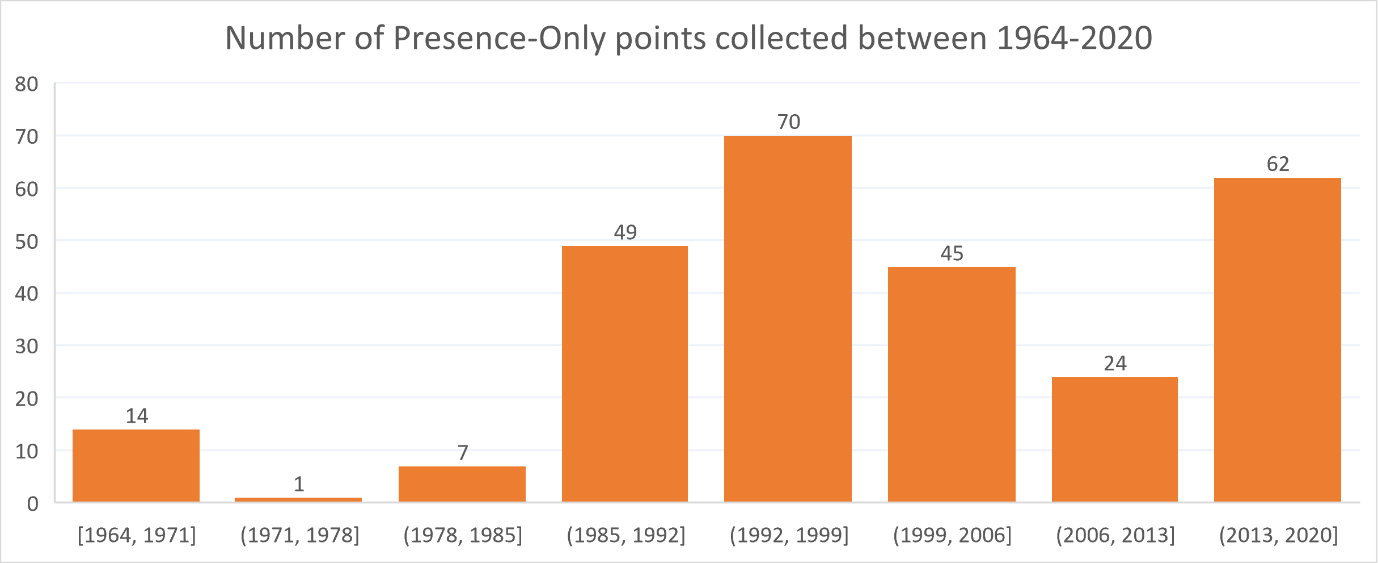
*

*Fig. 1: Number of presence records of Callithrix flaviceps collected between 1964 and 2020.
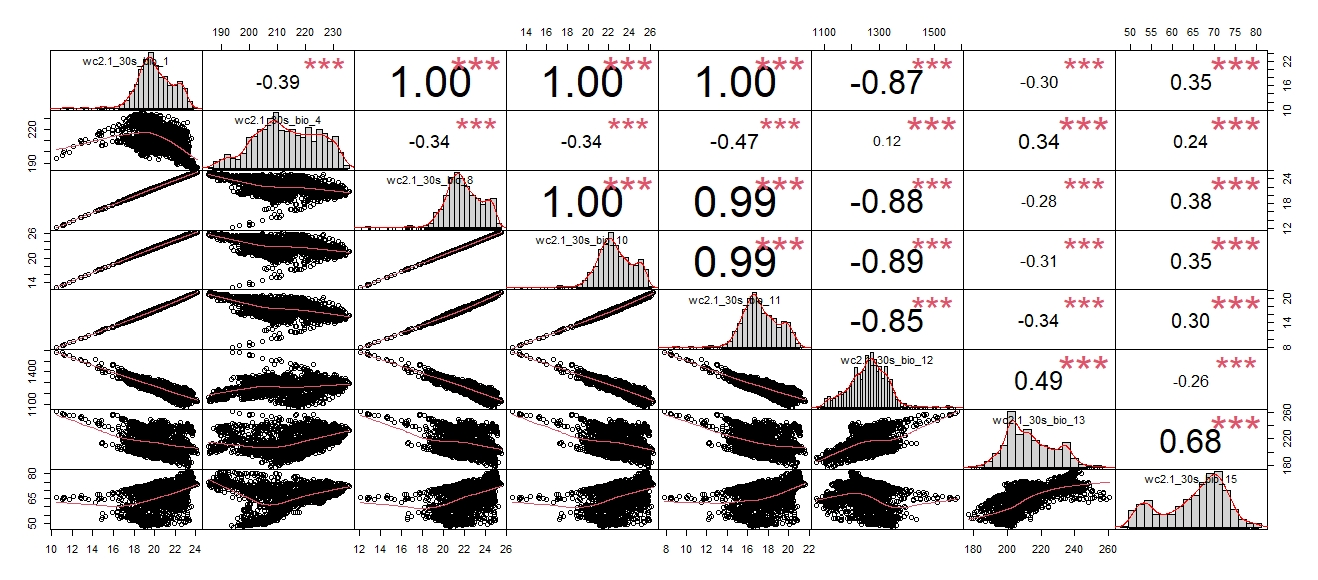
*

*Fig. 2: Correlation matrix between 8 bioclimatic variables for predicting current distribution of Callithrix flaviceps. wc2.1_30s_bio_1: Annual Mean Temperature, wc2.1_30s_bio_4: Temperature Seasonality (standard deviation ×100), wc2.1_30s_bio_8: Mean Temperature Wettest Quarter, wc2.1_30s_bio_10: Mean Temperature of Warmest Quarter, wc2.1_30s_bio_11: Mean Temperature of Coldest Quarter, wc2.1_30s_bio_12: Annual Precipitation, wc2.1_30s_bio_13: Precipitation of Wettest Month, wc2.1_30s_bio_15: Precipitation Seasonality (Coefficient of Variation).*


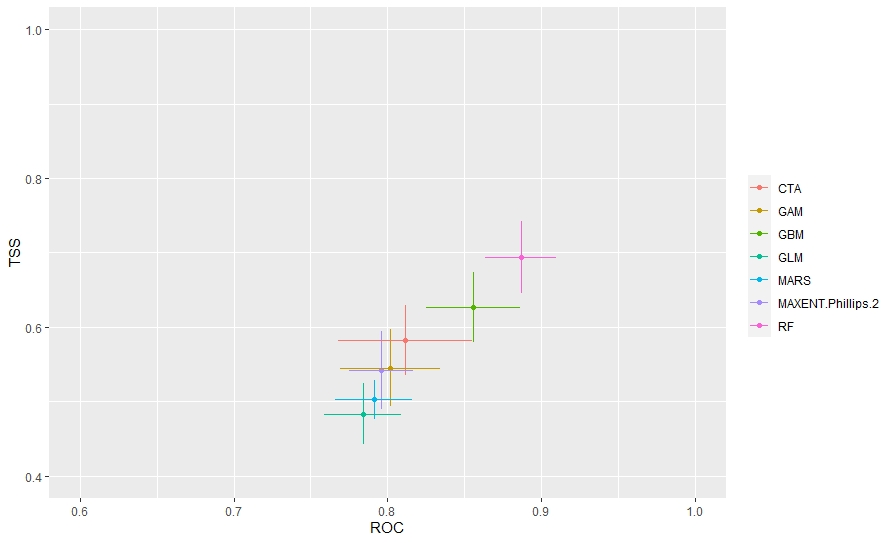


*Fig. 3: Model scores for each algorithm for predicting current species distribution of Callithrix flaviceps from climate and landscape variables according to the TSS (True Skill Statistics) and the ROC (Relative Operative Characteristic). Algorithms: CTA: Classification Tree Analysis, GAM: Generalised Additive Models, GBM: Generalised Boosting Models, GLM: Generalised Linear Models, MARS: Multivariate Adaptive Regression Splines, MAXENT: Maximum Entropy and RF: Random Forest. Error bars represent the range of the different models of the same algorithm, central point corresponds to average between models.*

*Table 2: Ensemble model scores. The mean, giving the mean probabilities across predictions; the weighted mean (wmean), estimating the weighted sum of probabilities; the committee averaging (ca), giving both a prediction and a measure of uncertainty, and the confidence interval (ci), showing 2 estimations (1 high and 1 low) of the confidence interval around the mean probability. ROC: Relative Operative Characteristic, TSS: True Skill Statistics.*

|  | ROC  testing data | TSS  testing data | | Sensitivity  (TSS) | Specificity  (TSS) |
| --- | --- | --- | --- | --- | --- |
| mean | 0.993 | 0.931 | 98.893 | | 94.220 |
| wmean | **0.993** | **0.931** | **99.262** | | **93.794** |
| ca | 0.989 | 0.945 | 99.262 | | 95.224 |
| ci inf | 0.983 | 0.877 | 96.679 | | 90.995 |
| ci sup | 0.993 | 0.945 | 100 | | 94.433 |

Part 1: Response curves for overall habitat suitability models

*
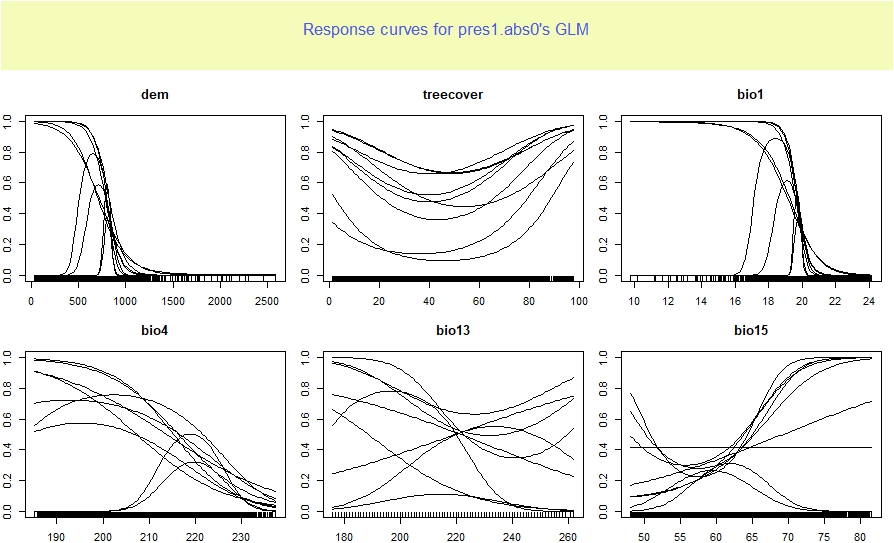
Fig. 4: Response Curves plot from generalised linear models (GLM). Where dem: Digital Elevation Model (m), treecover: Tree Canopy Cover (%), BIO1: Annual Mean Temperature (°C), BIO4: Temperature Seasonality (standard deviation*100), BIO13: Precipitation Wettest Month (mm), BIO15: Precipitation Seasonality (%). Each line represents a different model, with a different pseudo-absence selection and cross validation run.*


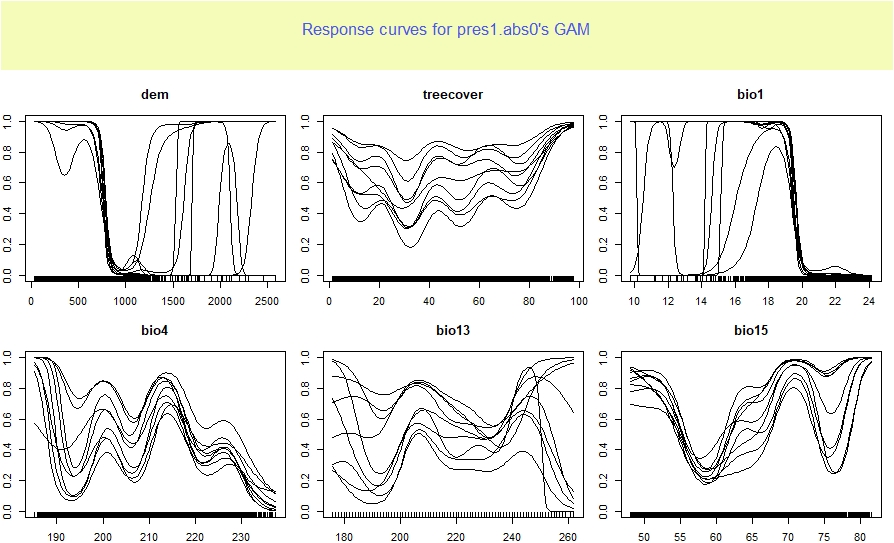


*Fig. 5: Response Curves plot from generalised additive models (GAM). Where dem: Digital Elevation Model (m), treecover: Tree Canopy Cover (%), BIO1: Annual Mean Temperature (°C), BIO4: Temperature Seasonality (standard deviation*100), BIO13: Precipitation Wettest Month (mm), BIO15: Precipitation Seasonality (%). Each line represents a different model, with a different pseudo-absence selection and cross validation run.*


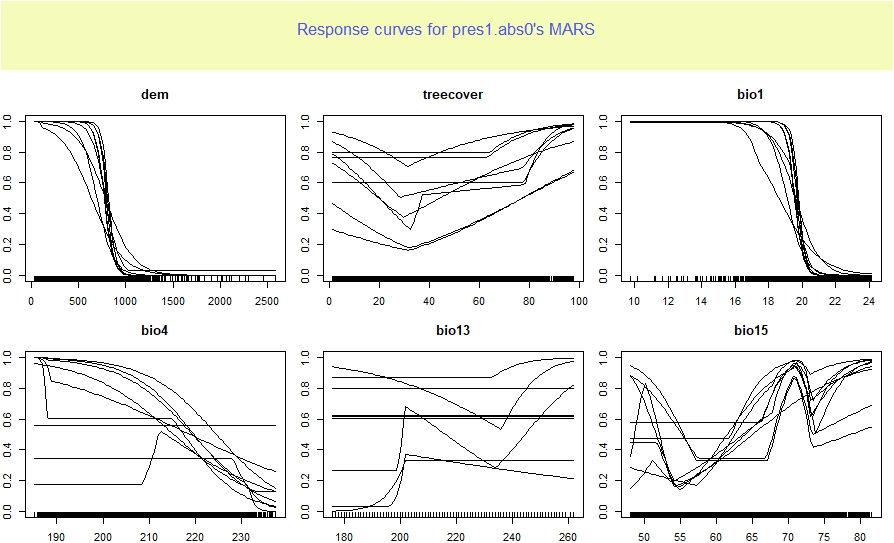


*Fig. 6: Response Curves plot from multivariate adaptive regression splines (MARS). Where dem: Digital Elevation Model (m), treecover: Tree Canopy Cover (%), BIO1: Annual Mean Temperature (°C), BIO4: Temperature Seasonality (standard deviation*100), BIO13: Precipitation Wettest Month (mm), BIO15: Precipitation Seasonality (%). Each line represents a different model, with a different pseudo-absence selection and cross validation run.*


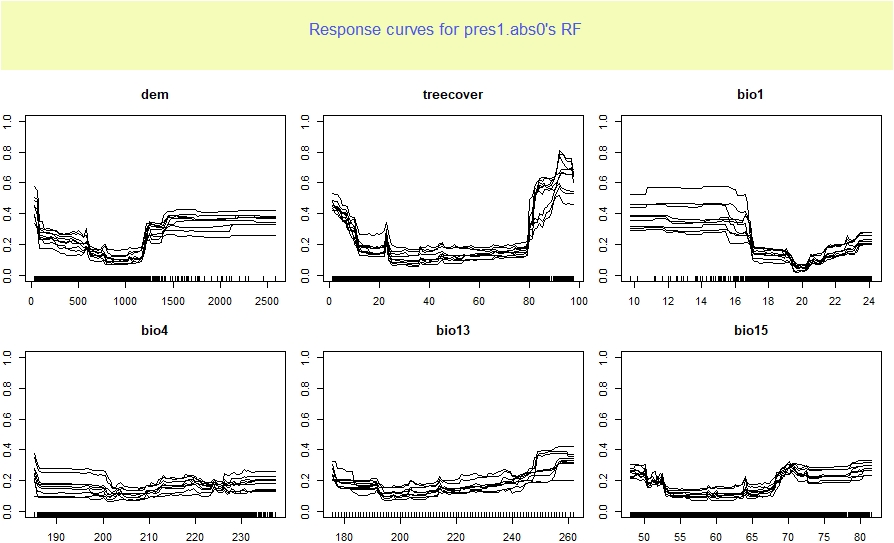


*Fig. 7: Response Curves plot from random forest (RF). Where dem: Digital Elevation Model (m), treecover: Tree Canopy Cover (%), BIO1: Annual Mean Temperature (°C), BIO4: Temperature Seasonality (standard deviation*100), BIO13: Precipitation Wettest Month (mm), BIO15: Precipitation Seasonality (%). Each line represents a different model, with a different pseudo-absence selection and cross validation run.*


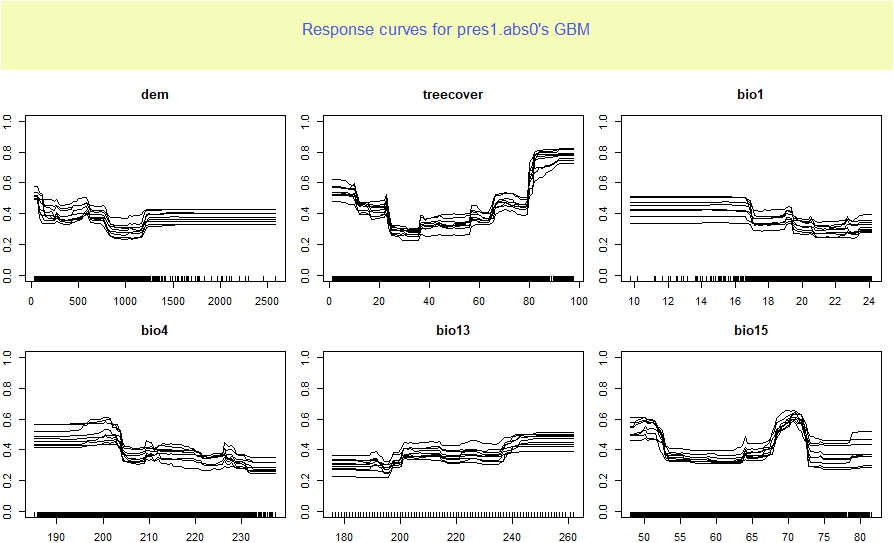


*Fig. 8: Response Curves plot from generalised boosting models (GBM). Where dem: Digital Elevation Model (m), treecover: Tree Canopy Cover (%), BIO1: Annual Mean Temperature (°C), BIO4: Temperature Seasonality (standard deviation*100), BIO13: Precipitation Wettest Month (mm), BIO15: Precipitation Seasonality (%). Each line represents a different model, with a different pseudo-absence selection and cross validation run.*


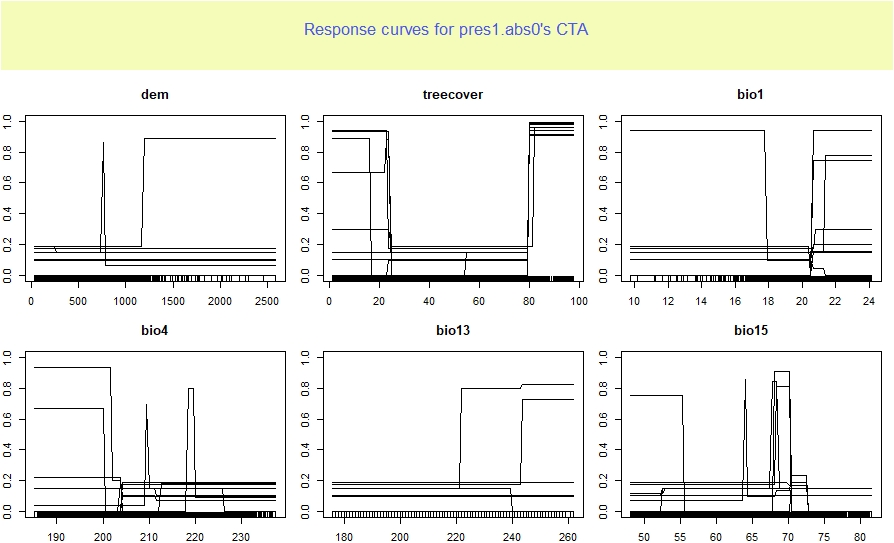


*Fig 9: Response Curves plot from classification tree analysis (CTA). Where dem: Digital Elevation Model (m), treecover: Tree Canopy Cover (%), BIO1: Annual Mean Temperature (°C), BIO4: Temperature Seasonality (standard deviation*100), BIO13: Precipitation Wettest Month (mm), BIO15: Precipitation Seasonality (%). Each line represents a different model, with a different pseudo-absence selection and cross validation run.*


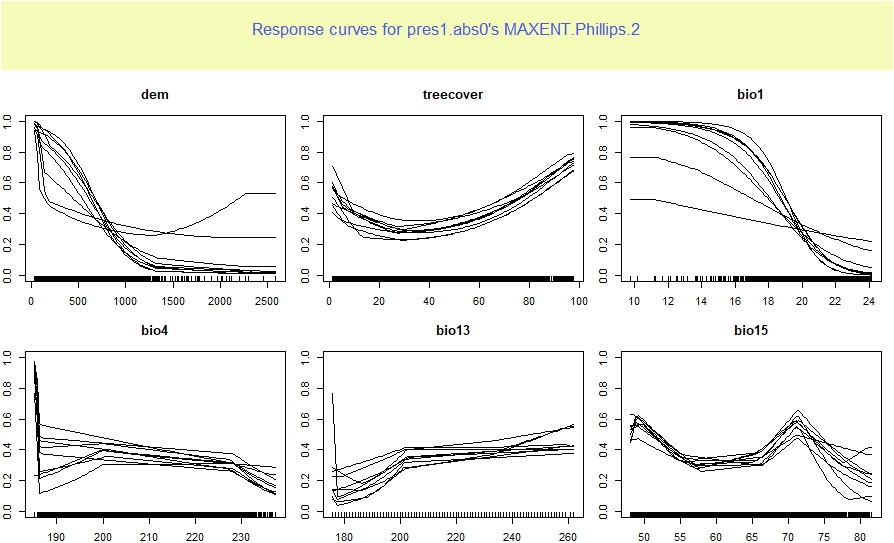


*Fig. 10: Response Curves plot from maximum entropy (MAXENT). Where dem: Digital Elevation Model (m), treecover: Tree Canopy Cover (%), BIO1: Annual Mean Temperature (°C), BIO4: Temperature Seasonality (standard deviation*100), BIO13: Precipitation Wettest Month (mm), BIO15: Precipitation Seasonality (%). Each line represents a different model, with a different pseudo-absence selection and cross validation run.*

Part 2: Response curves for climate only suitability models
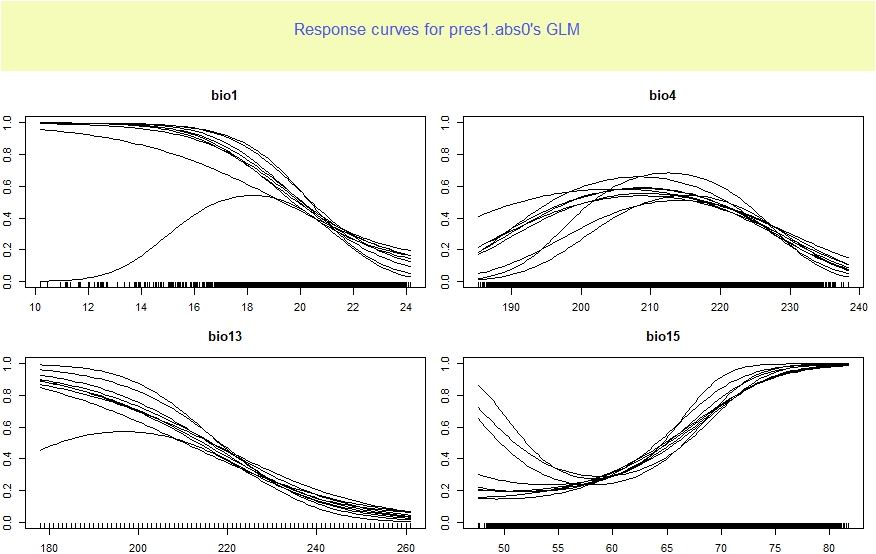


*Fig. 11: Response Curves for climate-only modelling, from generalised linear models (GLM). Where BIO1: Annual Mean Temperature (°C), BIO4: Temperature Seasonality (standard deviation*100), BIO13: Precipitation Wettest Month (mm), BIO15: Precipitation Seasonality (%). Each line represents a different model, with a different pseudo absence selection and cross validation run.*


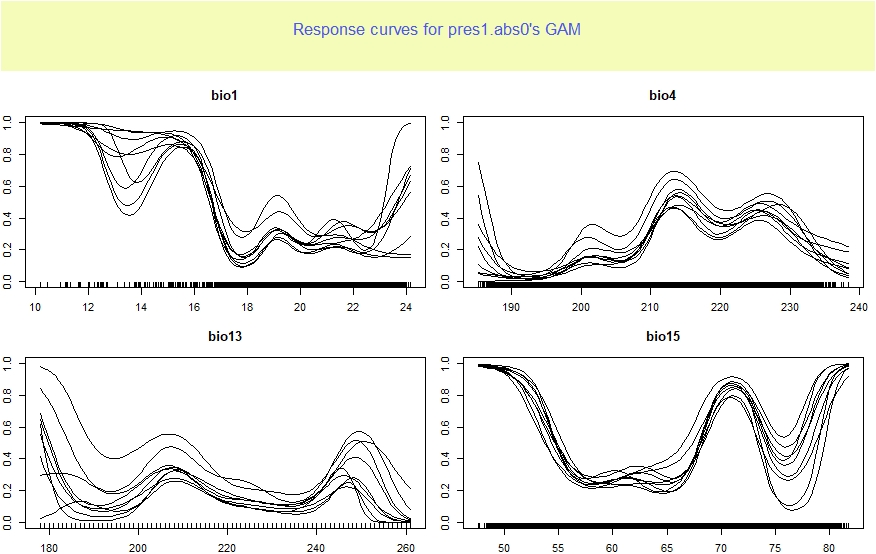


*Fig. 12: Response Curves for climate-only modelling from generalised additive models (GAM). Where BIO1: Annual Mean Temperature (°C), BIO4: Temperature Seasonality (standard deviation*100), BIO13: Precipitation
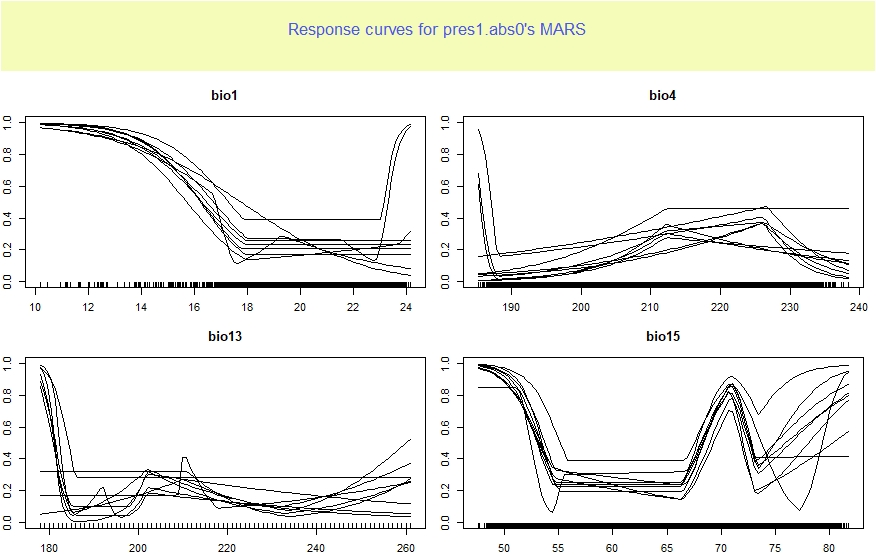
Wettest Month (mm), BIO15: Precipitation Seasonality (%). Each line represents a different model, with a different pseudo absence selection and cross validation run.*

*Fig. 13: Response Curves for climate-only modelling from multivariate adaptive regression spines (MARS). Where BIO1: Annual Mean Temperature (°C), BIO4: Temperature Seasonality (standard deviation*100), BIO13: Precipitation Wettest Month (mm), BIO15: Precipitation Seasonality (%). Each line represents a different model, with a different pseudo absence selection and cross validation run.*


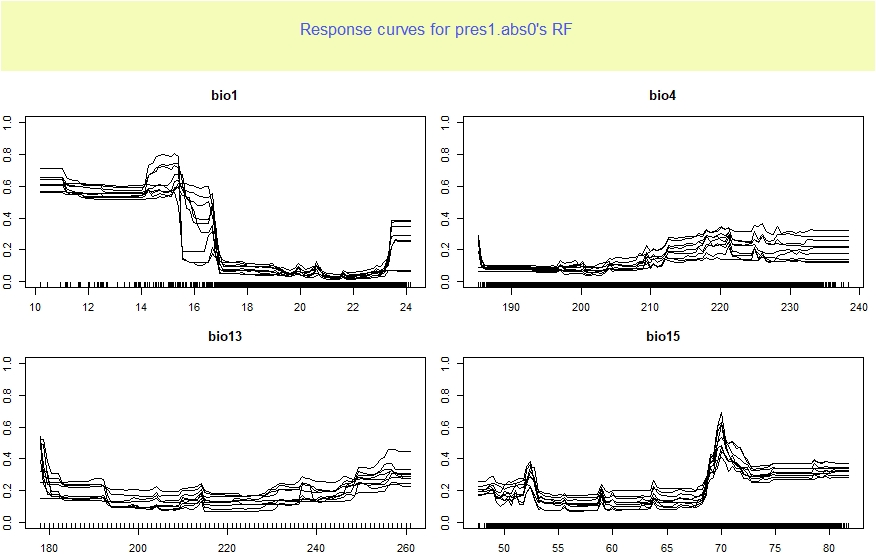


*Fig. 14: Response Curves for climate-only modelling from random forest (RF). Where BIO1: Annual Mean Temperature (°C), BIO4: Temperature Seasonality (standard deviation*100), BIO13: Precipitation Wettest Month (mm), BIO15: Precipitation Seasonality (%). Each line represents a different model, with a different pseudo absence selection and cross validation run.*


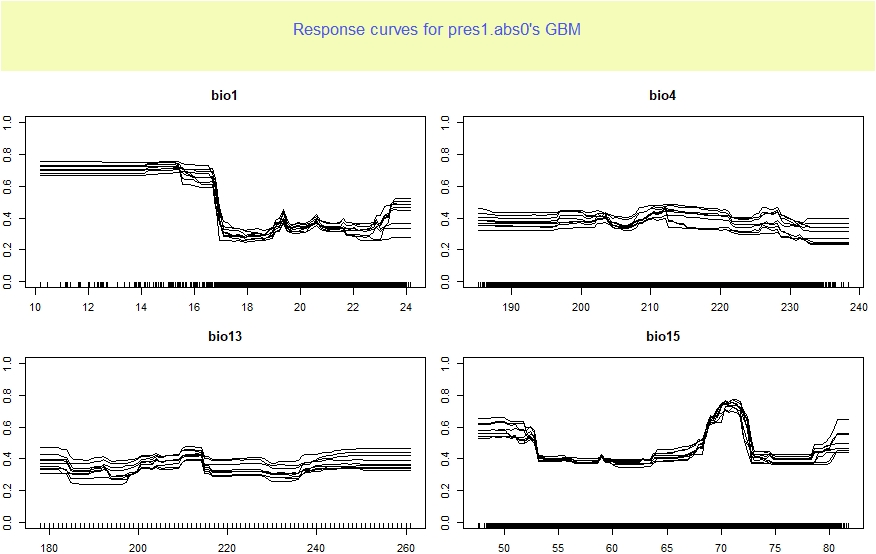


*Fig. 15: Response Curves for climate-only modelling, from generalised boosting models (GBM). Where BIO1: Annual Mean Temperature (°C), BIO4: Temperature Seasonality (standard deviation*100), BIO13: Precipitation Wettest Month (mm), BIO15: Precipitation Seasonality (%). Each line represents a different model, with a different pseudo absence selection and cross validation run.*


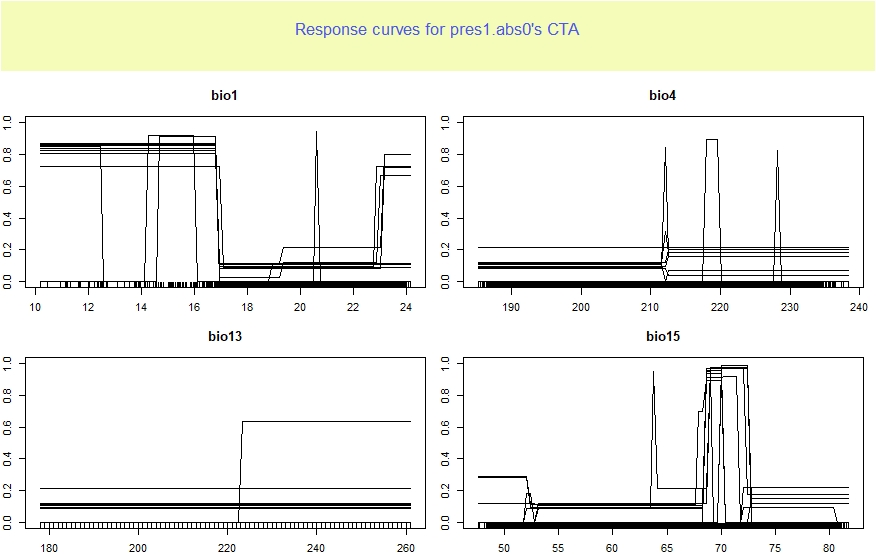


*Fig. 16: Response Curves for climate-only modelling, from classification tree analysis (CTA). Where BIO1: Annual Mean Temperature (°C), BIO4: Temperature Seasonality (standard deviation*100), BIO13: Precipitation Wettest Month (mm), BIO15: Precipitation Seasonality (%). Each line represents a different model, with a different pseudo absence selection and cross validation run.*


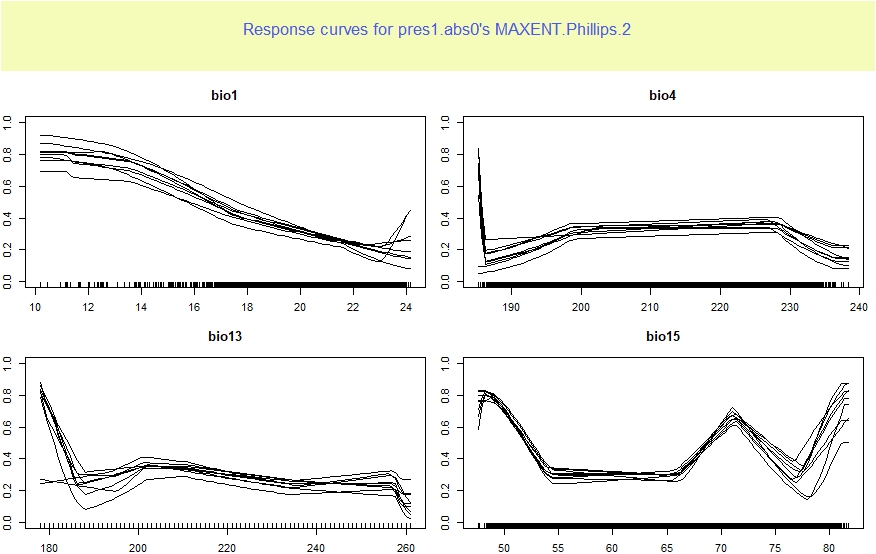


*Fig. 17: Response Curves for climate-only modelling, from maximum entropy (MAXENT). Where BIO1: Annual Mean Temperature (°C), BIO4: Temperature Seasonality (standard deviation*100), BIO13: Precipitation Wettest Month (mm), BIO15: Precipitation Seasonality (%). Each line represents a different model, with a different pseudo absence selection and cross validation run.*

Part 3: Response curves for landscape only suitability models


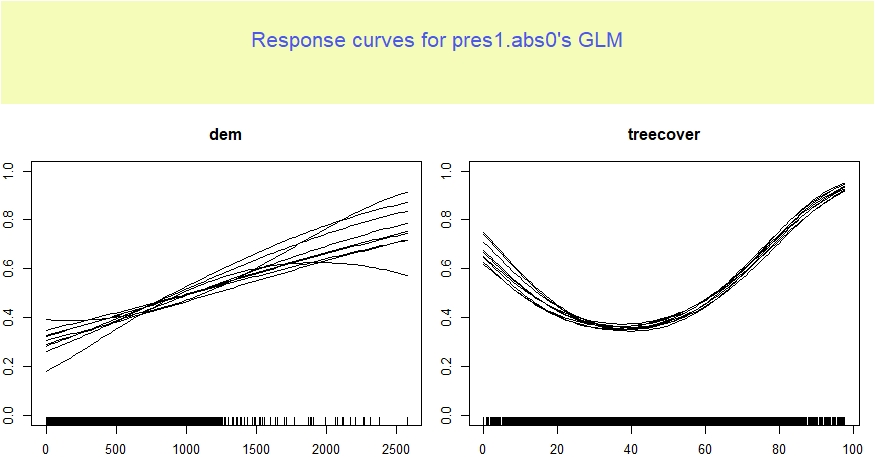


*Fig. 18: Response Curves plot from generalised linear models (GLM). Where dem: Digital Elevation Model (m) and treecover: Tree Canopy Cover (%). Each line represents a different model, with a different pseudo absence selection and cross validation run.*


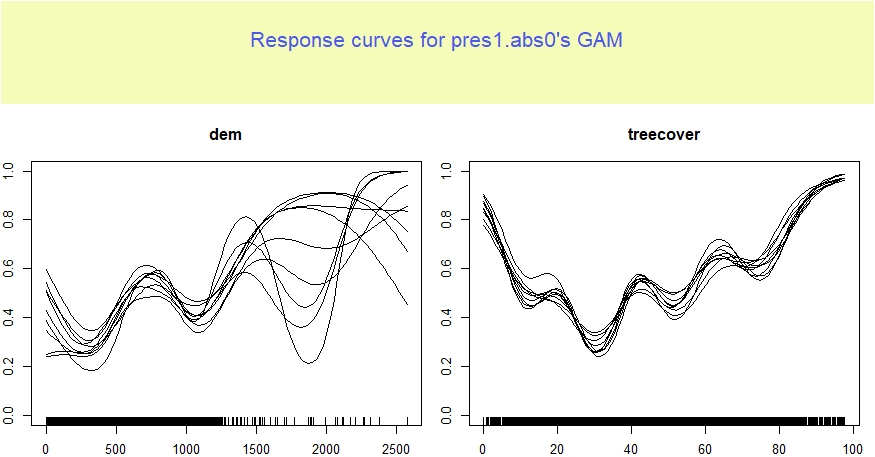


*Fig. 19: Response Curves plot from generalised additive models (GAM). Where dem: Digital Elevation Model (m) and treecover: Tree Canopy Cover (%). Each line represents a different model, with a different pseudo absence selection and cross validation run.*


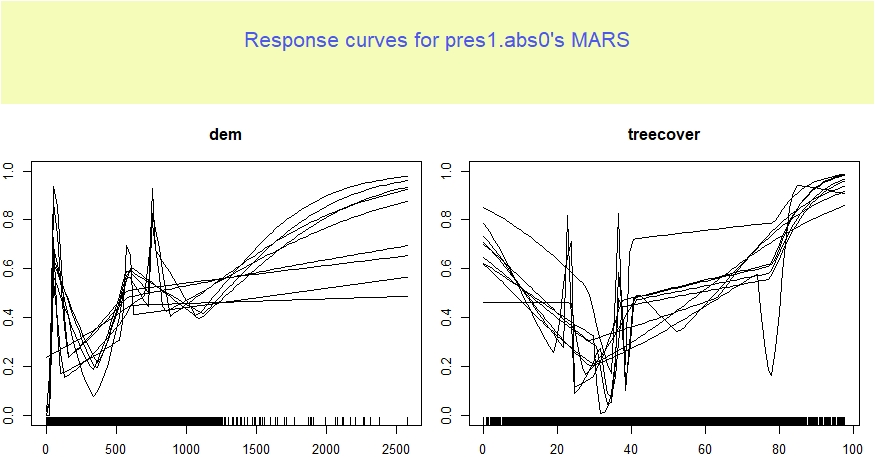


*Fig. 20: Response Curves plot from multivariate adaptive regression spline (MARS). Where dem: Digital Elevation Model (m) and treecover: Tree Canopy Cover (%). Each line represents a different model, with a different pseudo absence selection and cross validation run.*


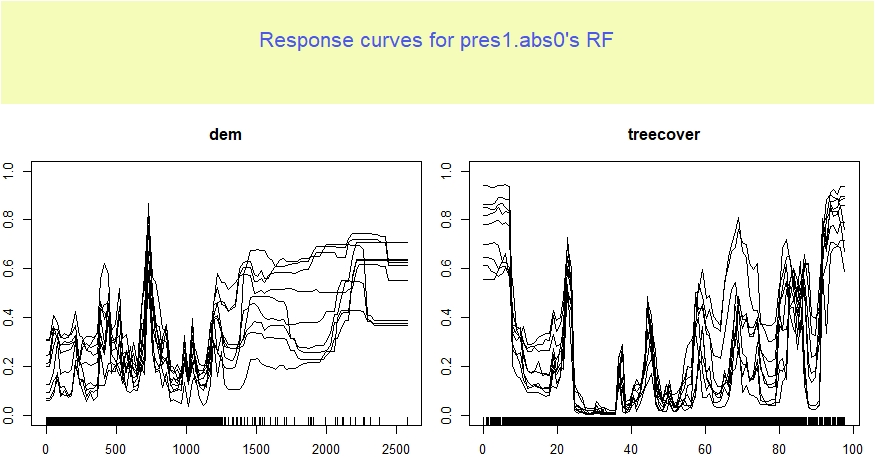


*Fig. 21: Response Curves plot from random forest (RF). Where dem: Digital Elevation Model (m) and treecover: Tree Canopy Cover (%). Each line represents a different model, with a different pseudo absence selection and cross validation run.*


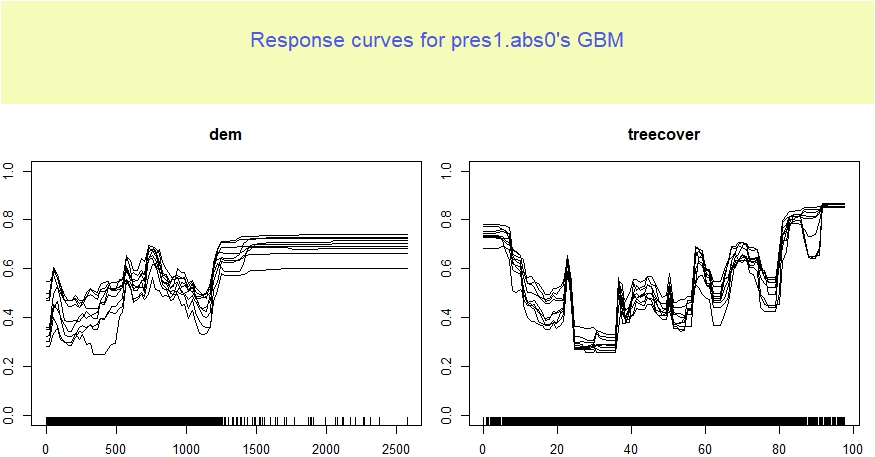


*Fig. 22: Response Curves plot from generalised boosting models (GBM). Where dem: Digital Elevation Model (m) and treecover: Tree Canopy Cover (%). Each line represents a different model, with a different pseudo absence selection and cross validation run.*


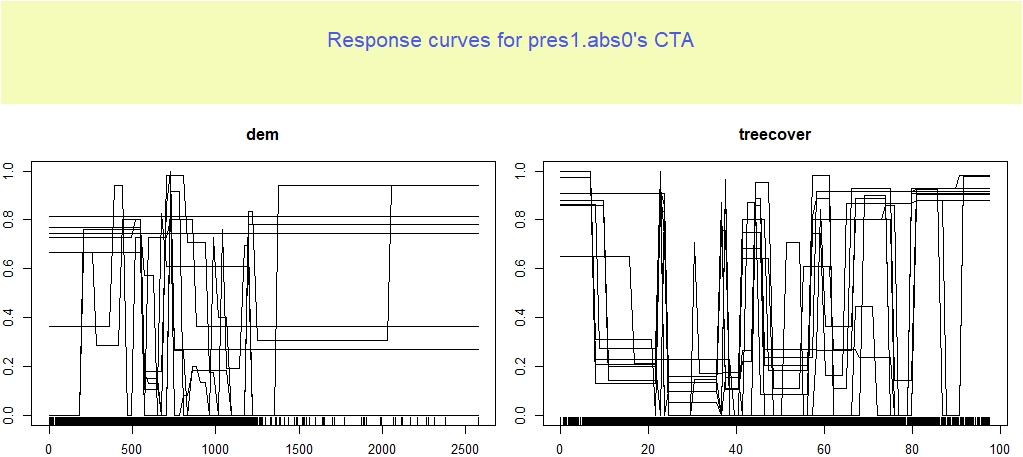


*Fig. 23: Response Curves plot from classification tree analysis (CTA). Where dem: Digital Elevation Model (m) and treecover: Tree Canopy Cover (%). Each line represents a different model, with a different pseudo absence selection and cross validation run.*


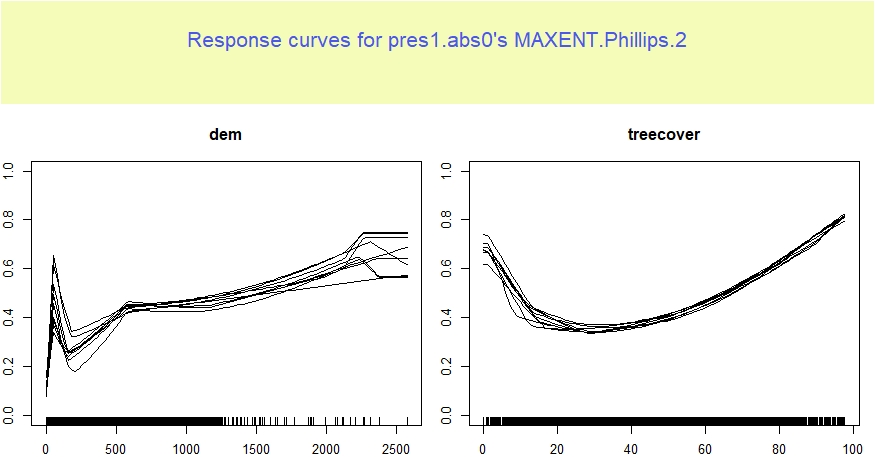


*Fig. 24: Response Curves plot from maximum entropy (MAXENT). Where dem: Digital Elevation Model (m) and treecover: Tree Canopy Cover (%). Each line represents a different model, with a different pseudo absence selection and cross validation run.*

Part 4: Variable importance according to climate only and landscape-only modelling and current suitability

*
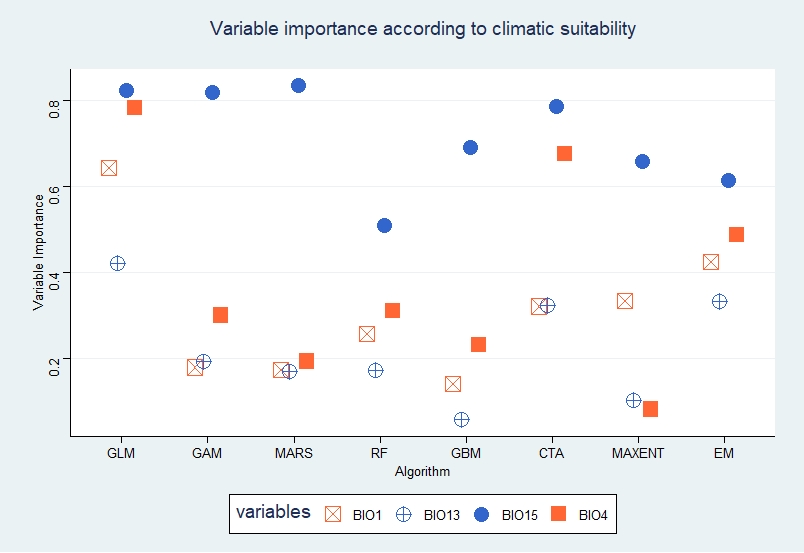
*

*Fig. 25: Mean variable importance for each algorithm and ensemble models for predicting current species distribution of Callithrix flaviceps. Values are relative to each other, values closer to 1 are considered more important for predicting the species distribution. Climatic variables: BIO1 (Annual Mean Temperature), BIO4 (Temperature Seasonality), BIO13 (Precipitation of Wettest Month), BIO15 (Precipitation Seasonality). Algorithms: GLM: Generalised Linear Models; GAM: Generalised Additive Models, MARS: Multivariate Adaptive Regression Splines, RF: Random Forest, GBM: Generalised Boosting Models, CTA: Classification Tree Analysis, MAXENT: Maximum Entropy, EM: Ensemble Models.*

*
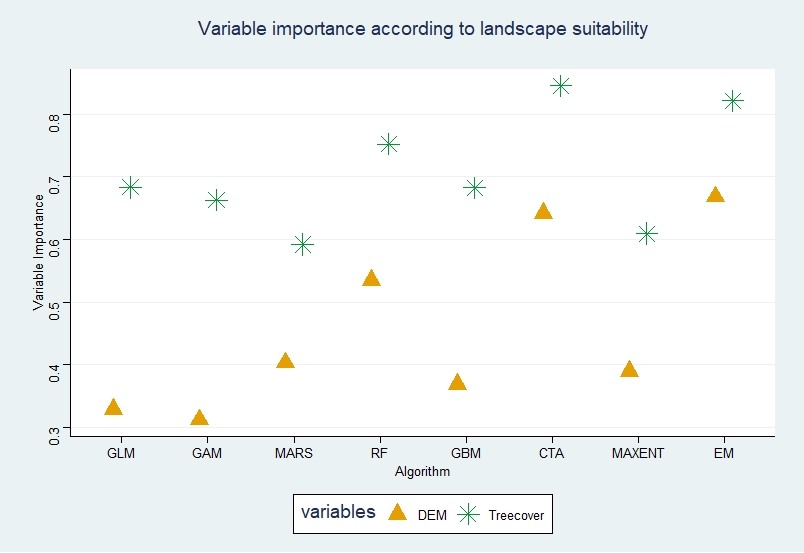
*

*Fig. 26: Mean of variable importance for each algorithm and ensemble models for predicting current species distribution of Callithrix flaviceps. Values are relative to each other, values closer to 1 are considered more important for predicting the species distribution. DEM (Digital Elevation Model), Treecover (Tree Canopy Cover). GLM: Generalised Linear Models; GAM: Generalised Additive Models, MARS: Multivariate Adaptive Regression Splines, RF: Random Forest, GBM: Generalised Boosting Models, CTA: Classification Tree Analysis,
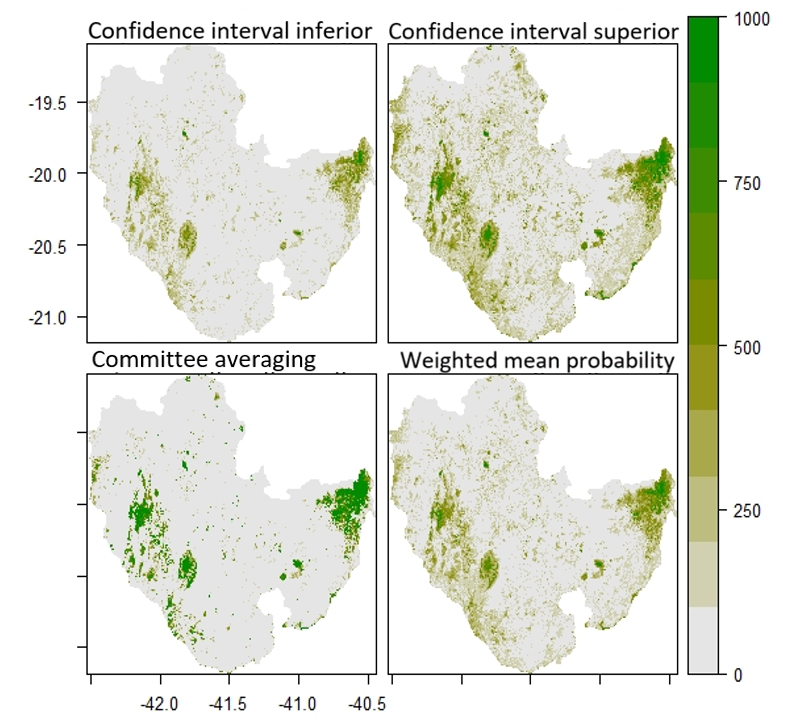
MAXENT: Maximum Entropy, EM: Ensemble Models.*

*Fig. 27: Current prediction of overall habitat suitability for Callithrix flaviceps. Each map represents a different Ensemble Model. The 2 tops maps show 2 levels of confidence interval around the mean probability. The committee averaging shows the consistency between models, value of 1 (dark green) means that all models agreed to predict presence, while value of 0 (grey) means that all models agreed to predict absence of the species, yellow/light green shows inconsistency between models. The weighted mean probability represents the actual projection, dark green represents high suitable areas.*

Part 5: Future predictions for climate and landscape only modelling

*
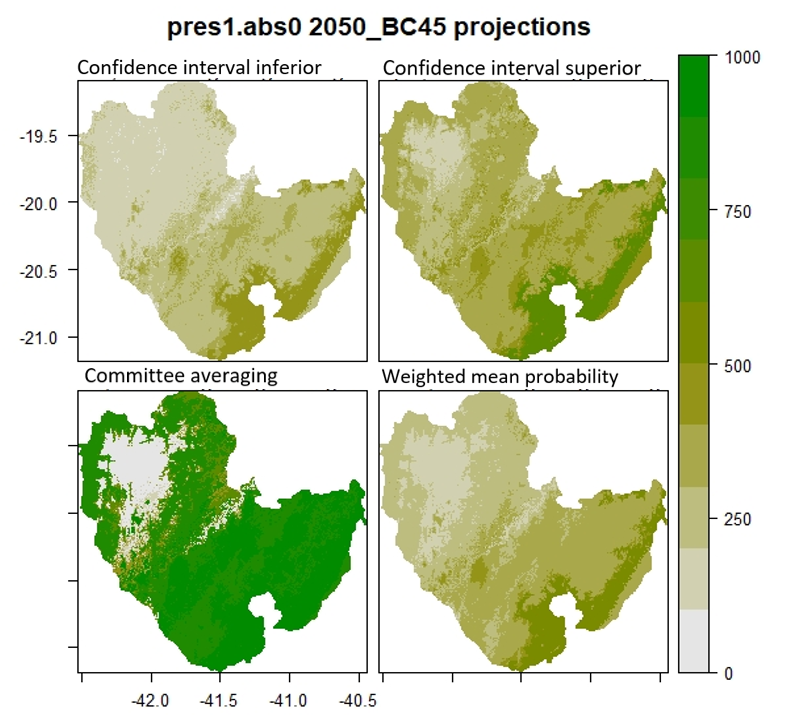
Fig. 28: Future (2050) prediction of climatic suitability for Callithrix flaviceps under climate scenario RCP45. Each map represents a different Ensemble Model. The 2 tops maps show 2 levels of confidence interval around the mean probability. The committee averaging shows the consistency between models: dark green indicates that all models agreed to predict presence, whereas grey indicates that all models agreed to predict absence of the species; yellow/light green shows inconsistencies between model predictions. The weighted mean probability represents the actual ensemble prediction, with dark green representing highly suitable areas.*

*
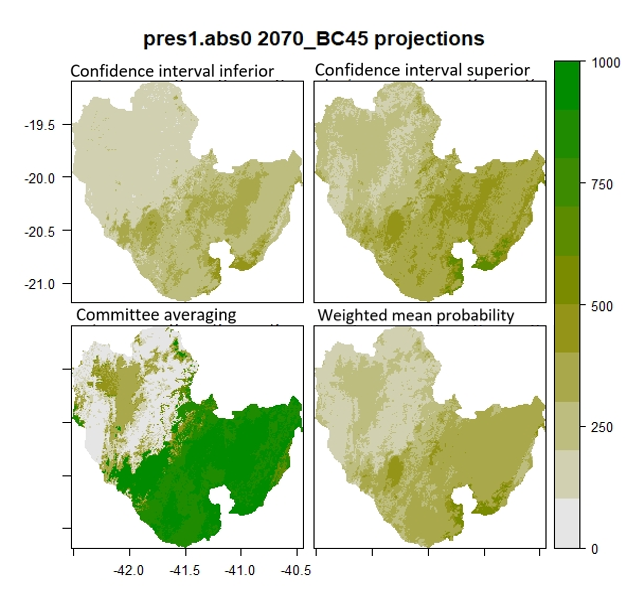
*

*Fig. 29: Future (2070) prediction of climatic suitability for Callithrix flaviceps under climate scenario RCP45. Each map represents a different Ensemble Model. The 2 tops maps show 2 levels of confidence interval around the mean probability. The committee averaging shows the consistency between models: dark green indicates that all models agreed to predict presence, whereas grey indicates that all models agreed to predict absence of the species; yellow/light green shows inconsistencies between model predictions. The weighted mean probability represents the actual ensemble prediction, with dark green representing highly suitable areas.*


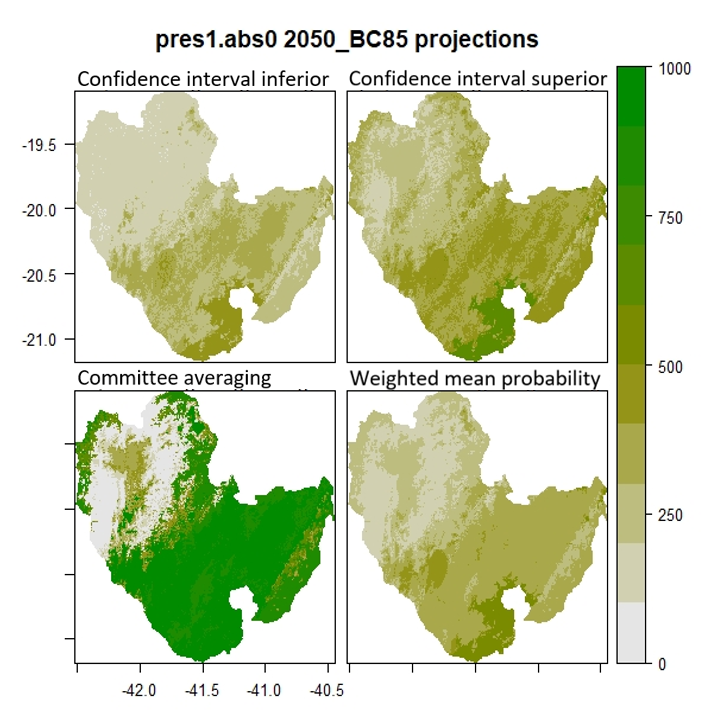


*Fig. 30: Future (2050) prediction of climatic suitability for Callithrix flaviceps under climate scenario RCP85. Each map represents a different Ensemble Model. The 2 tops maps show 2 levels of confidence interval around the mean probability. The committee averaging shows the consistency between models: dark green indicates that all models agreed to predict presence, whereas grey indicates that all models agreed to predict absence of the species; yellow/light green shows inconsistencies between model predictions. The weighted mean probability represents the actual ensemble prediction, with dark green representing highly suitable areas.*


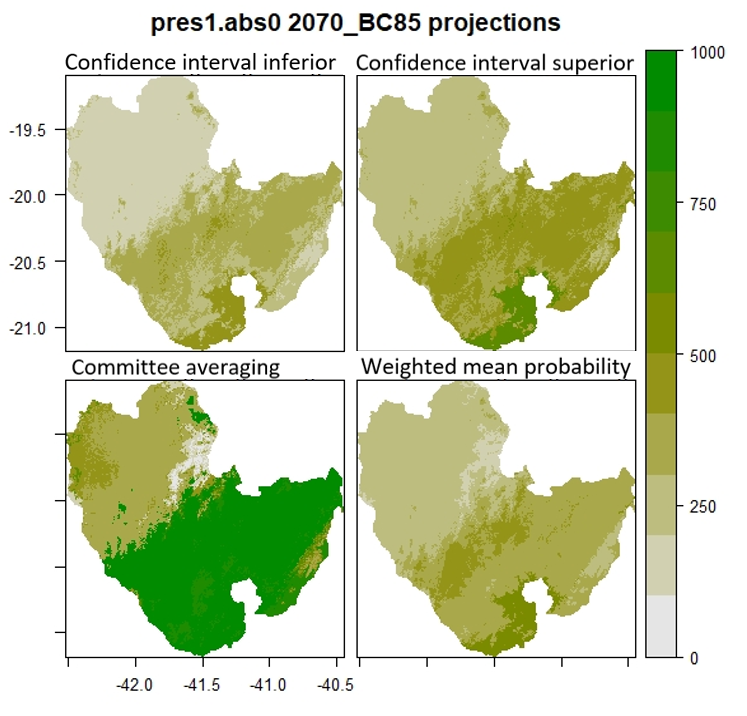


*Fig. 31: Future (2070) prediction of climatic suitability for Callithrix flaviceps under climate scenario RCP85. Each map represents a different Ensemble Model. The 2 tops maps show 2 levels of confidence interval around the mean probability. The committee averaging shows the consistency between models: dark green indicates that all models agreed to predict presence, whereas grey indicates that all models agreed to predict absence of the species; yellow/light green shows inconsistencies between model predictions. The weighted mean probability represents the actual ensemble prediction, with dark green representing highly suitable areas.*
